# Sugars to Acids via Thioesters: A Computational Study

**DOI:** 10.3390/life15081189

**Published:** 2025-07-26

**Authors:** Jeremy Kua, Jonathan D. Karin

**Affiliations:** Department of Chemistry & Biochemistry, University of San Diego, San Diego, CA 92110, USA

**Keywords:** origins of life, prebiotic chemistry, thioester, proto-metabolism

## Abstract

Extant core metabolic cycles such as the TCA cycle and its related analog pathways utilize carboxylic acids as metabolites, with thioesters playing a key role. We examine if sugars from the potentially autocatalytic formose reaction can be converted to carboxylic acids in the absence of enzymes by calculating the thermodynamics and kinetics of such pathways. We zero in on a mechanism involving the addition of a thiol to an aldehyde, followed by intramolecular disproportionation to form a thioester that can be hydrolyzed into its carboxylic acid. This route is thermodynamically favorable but can have kinetic bottlenecks. We find that elimination of H_2_O or H_2_S is often the rate-determining step, and that alpha di-carbonyl reactants that do not require such a step are more feasible in the absence of catalysts.

## 1. Introduction

The core of extant metabolism essentially involves metabolites containing just carbon, hydrogen and oxygen; in the tricarboxylic acid (TCA) cycle and its closely related analog pathways (such as the 3HP/4HB cycle), these metabolites are carboxylic acids [1]. When run in the oxidative direction, these metabolic cycles are catalytic and feature highly evolved enzymes that lower the otherwise high reaction barriers. However, if run in the reverse direction, the cycle is potentially autocatalytic, and the net overall reaction formally converts two CO_2_ into an acetate analog, i.e., C_1_ + C_1_ → C_2_. While autocatalytic cycles are common in extant metabolism [2], they are rarely observed in simple benchtop chemistry. One rare example, often invoked in discussions on the chemical origins of life and prebiotic chemistry, is the formose reaction. Starting from formaldehyde (CH_2_O) as the C_1_ species, a plethora of sugars is produced in a complex messy reaction [3] that also includes carboxylic acids and polyols as side-products.

**Figure 1:**
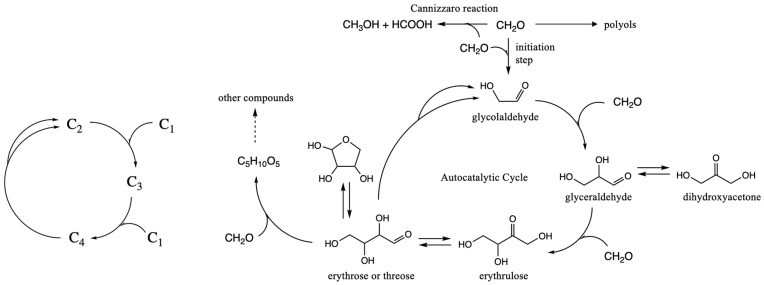
Core autocatalytic cycle of the formose reaction. Reproduced from ref. [22] with permission from the authors.

The mechanisms of the formose reaction have been well-studied [4,5,6]. The smallest cycle involves just three reactions: (1) C_2_ + C_1_ → C_3_ aldol addition, (2) C_3_ + C_1_ → C_4_ aldol addition, and (3) C_4_ → C_2_ + C_2_ retro-aldol reaction, as shown schematically in the lefthand side of Figure 1. If only C_1_ is present, the reaction is very slow. The direct addition of two C_1_ species to form C_2_ is kinetically very challenging because there is no umpolung species to favor C–C bond formation. (This is true for CH_2_O in the formose reaction with its partially positive carbon, and even more so for CO_2_ in extant metabolism.) The presence of even a small amount of the key C_2_ molecule, glycolaldehyde, is sufficient to trigger the autocatalytic cycle, because the aldol additions have significantly lower barriers with enolizable aldehydes present. (A small amount of the C_3_ or C_4_ species is also sufficient to bypass the unfavorable direct C_1_ + C_1_ → C_2_ reaction.) As shown in Figure 1, the complexity of the formose reaction with its wide suite of products is because larger sugars can be formed by further aldol additions (especially as C_1_ “food” runs out). Furthermore, the Cannizzaro reaction that disproportionates aldehydes into carboxylic acids and alcohols is always present. Changing the reaction conditions can lead to different suites of products, as shown by the Huck group in a series of systematic studies [7,8,9].

How might the formose cycle involving sugars transition into a proto-metabolic cycle with carboxylic acids as proto-metabolites? Why is acetate the key C_2_ species in extant metabolic cycles? Why are thioester species such as acetyl-CoA part of such cycles? The salient presence of thioesters led De Duve to propose a prebiotic “thioester world” [10] whereby thioesters may have played an early role in energy transduction when phosphate species were locked up in minerals and not easily available in solution [11,12], although there are recent suggestions that dissolved Fe^2+^ may increase phosphate availability [13], or that reduced phosphorus compounds may have been the precursors [14,15]. Analysis of biochemical networks suggests that the most ancient metabolic pathways may have been phosphate-free, and intriguingly organosulfur compounds are featured in their place [16,17]. Sulfur’s role at the origin of life has been spotlighted in Wachterhauser’s “pyrite world” [18] and Sutherland’s “cyanosulfidic world” [19]. Sulfur’s broad role in prebiotic chemistry was recently reviewed by Vallee et al. [20] and earlier this year Devaraj et al. published work utilizing thioesters to create protocells [21].

Our group is interested in the role of sulfur at the origin-of-life. In generating a thermodynamic map of the free energies of small CHOS compounds, we found that thiols are stabilized compared to their hydroxyl counterparts [22]. We followed up with a detailed analysis of the formose reaction with mercaptoaldehyde (rather than glycolaldehyde) as the key C_2_ species [23], finding that the presence of thiol groups lowered crucial kinetic barriers leading to a thermodynamically more favorable C_4_ → C_2_ + C_2_ retro-aldol reaction. Thus, a formose-like core autocatalytic cycle containing sulfur analogs with a higher turnover rate could potentially outcompete one that lacked sulfur. Our results also suggested that high H_2_S concentrations (and therefore higher thiol concentration) kinetically disfavored the parasitic Cannizzaro reaction.

The current work investigates if the presence of thiols could facilitate the transition from sugars to acids. Thioesters are a key intermediate in this pathway, and their formation is significantly exergonic providing a driving force for this reaction. We took inspiration from work by Weber and co-workers in the 1980s that unfortunately was not followed-up by other experimental groups. Weber had proposed the formation of “energy-rich thioesters” by reacting glyceraldehyde with N-acetylcysteine [24]. Experimentally, the synthesis led to a lactic acid thioester suggesting that an intramolecular disproportionation is a crucial step in the transformation [25]; although Cannizzaro side-products are also observed under their reaction conditions.

Utilizing computational chemistry, we calculate the thermodynamics and kinetics for transforming small sugars (CH_2_O)_n_ for n = 2–4 into their corresponding thioesters. We find that hydrolysis of the thioesters is exergonic by 5–7 kcal/mol; intriguingly, this value is similar to ATP hydrolysis and may support De Duve’s thioester world hypothesis. Weber had also shown that formation of pyrophosphate is facilitated by thioesters providing a direct link between the two energy “currencies” [26,27]. Our chosen sugar reactants come from the core of the formose cycle and its corresponding sulfur analogs. We find that elimination of H_2_O or H_2_S is often the rate-determining step; and reactants such as alpha di-carbonyl compounds do not require such a step as part of the intramolecular disproportionation are kinetically more feasible.

Before getting into the results and discussion, we describe our computational protocol and its limitations. Our current work examines in detail the reaction pathways analogous to Weber’s reaction (albeit with CH_3_SH as the thiol in our computations rather than a cysteine analog) but goes beyond it by explicitly connecting formose sugars (and their dicarbonyl analogs) to thioesters that when hydrolyzed lead to extant metabolic compounds. Our proposed intramolecular disproportionation mechanism fleshes out in detail what is hinted at by Weber, including multiple intermediates not easily isolated experimentally, and proposes where the bottlenecks might be. The scope of our study does not include other recent experimental work on thioesters in prebiotic chemistry; and we would like to point readers interested in the scope of thioesters at the origin of life to work by the Vallee lab [28,29] and Frenkel-Pinter et al. [30]. Nor have we included thioester-facilitated carboxylation mechanisms proposed by De Duve, which will be the subject of a future investigation.

## 2. Computational Methods

We use the same computational protocol as our recent work on CHOS molecules and sulfur analogs of the formose cycle. This allows us to make direct comparisons and extend our free energy map. Here, we provide a brief description of that protocol for the convenience of our readers. Much of the text in this section (and the first two paragraphs of the next section) is reproduced from those two articles (published in this journal) [22,23] since we think the description is both clear and succinct. Our calculated free energies using this quantum chemistry protocol showed good agreement with available experimental results for CHO systems [31,32,33,34].

Here are the computational details: The geometry of each molecule is optimized and its electronic energy calculated at the B3LYP (Becke3-Lee-Yang-Parr) [35,36,37,38] flavor of density functional theory with the 6-311G** basis set. To maximize the probability of finding global minima, multiple conformers are generated using molecular mechanics (MMFFs force field [39]). The optimized structures are embedded in a continuum dielectric to calculate the aqueous solvation contribution to the free energy. While this does not provide a specific concentration, it assumes a dilute solution such that the electrostatic field generated by a neighboring solute molecule is effectively screened by the water solvent. One can consider all solutes to have the same relative concentrations in our calculations. Since our previous work [23] found a handful of transition states, for uniformity, we used the SMD (Solvation Model based on Density) implicit solvent [40] in all transition state calculations. (There was practically no difference in the solvation free energy for minima).

Zero-point energy corrections are included, and we apply the standard temperature-dependent enthalpy correction term (for 298.15 K) from statistical mechanics by assuming translational and rotational corrections are a constant times *kT*, and that low frequency vibrational modes generally cancel out when calculating enthalpy differences. However, entropic corrections in aqueous solution are problematic [41,42,43]. Changes in free energy terms for translation and rotation are poorly defined in solution due to restricted complex motion, particularly as the size of the molecule increases (thus increasing its conformational entropy). Free energy corrections come from two different sources: thermal corrections and implicit solvent. Neither of these parameters is easily separable, nor do they constitute all the required parts of the free energy. We follow the approach of Deubel & Lau [44], assigning the solvation entropy of each species as *half* its gas-phase entropy (calculated using standard statistical mechanics approximations similar to the enthalpy calculations described above), based on proposals by Wertz [45] and Abraham [46] that upon dissolving in water, molecules lose a constant fraction (~0.5) of their entropy.

To estimate activation energies, transition states were optimized by including several explicit water and/or catalytic molecules to aid transferring H moieties. All calculated transition states have one significant negative eigenvalue corresponding to the reaction coordinate, and each was checked to ensure it had the correct eigenvectors for bond breaking/forming connecting the transition state to its reactants and products. Several conformers built by hand are tested in each case and we only report the lowest calculated barriers with the optimum number of solvent molecules added to assist in proton transfer.

Our protocol performs well comparing the equilibrium concentrations in a self-oligomerizing solution of 1 M glycolaldehyde at 298 K to subsequent NMR measurements [33]. Our relative Gibbs free energies in aqueous solution are typically within 0.5 kcal/mol compared to experiment. That being said, our protocol has systematic errors of 2–3 kcal/mol when calculating barriers involving carbonyl chemistry when compared to experimental results. Going to a higher level of theory does not reduce this error [47]; nor does using anionic species and our protocol actually does better when carboxylic acids are calculated in their neutral COOH form [34]. While protonation may vary with pH, we expect thermodynamic trends not to vary significantly within plausibly prebiotic conditions of pH 5–9 from our reference state, since almost all the compounds in our pathways remain in their neutral form in this pH range, with the exception of the final carboxylic acids (not part of the route from sugar to thioester). The kinetics however may change. There are also specific computational problems with including cations in our protocol as discussed in previous work [48,49] which also showed how to capture systematic changes in activation energies using a range of neutral catalysts in our protocol. Quantum chemistry is about error cancelation, and our protocol (with its foibles, including the simplistic entropy correction) has worked well even with a systematic error for activation barriers.

## 3. Results and Discussion

To connect this work to our prior CHOS thermodynamic maps [22,23], we use the same set of reference compounds: CO_2_, H_2_, H_2_O and H_2_S are assigned a *relative* free energy, *G*_rel_ of 0.0 kcal/mol. The *G*_rel_ values of all other species can be determined by calculating the change in free energy for forming the species, analogous to a free energy of formation. For example, the formation reaction of mercaptoaldehyde (C_2_H_4_OS) is written as

2 CO_2_ + 4 H_2_ + H_2_S → C_2_H_4_OS + 3 H_2_O

Since Δ*G* of this reaction is −6.2 kcal/mol, we assign *G*_rel_(C_2_H_4_OS) = −6.2 kcal. For the rest of this paper, we will use the unit kcal as shorthand to signify kcal/mol. The *G*_rel_ values for all compounds parsed into their separate enthalpic, entropic, and solvation free energy are provided in Supplementary Information.

A consistent set of reference compounds allows us to globally compare energies. In the Figures, *G*_rel_ values are found next to each compound for local minima and in square brackets next to an arrow for transition states. We use Δ*G* to designate the *change* in free energy when focusing on a particular reaction, where Δ*G* = *G*_rel_(products) − *G*_rel_(reactants). Similarly, when we refer to the barrier of a specific reaction, we designate this Δ*G*^‡^ which compares *G*_rel_ of the transition state to either the reactants or products depending on whether the forward or reverse reaction is being discussed. Note that several reactions involve the non-reference compound CH_3_SH which has a *G*_rel_ of −19.2 kcal.

### 3.1. Glycolaldehyde and Mercaptoaldehyde

**Figure 2:**
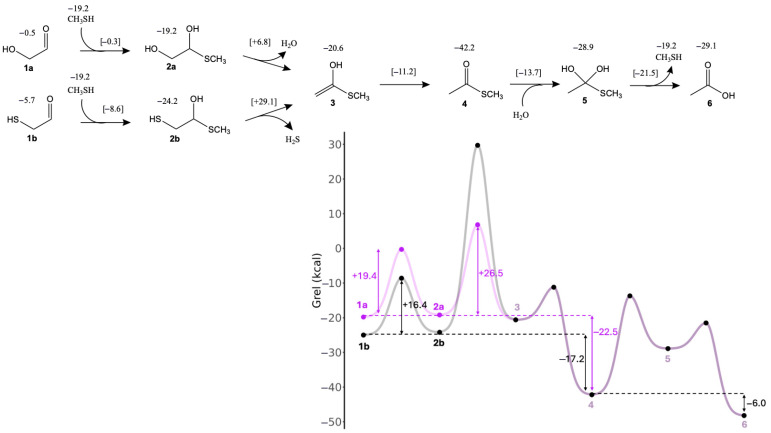
Formation of acetic acid and thioester from C_2_ aldehyde (*G*_rel_ in kcal).

**Figure 3:**
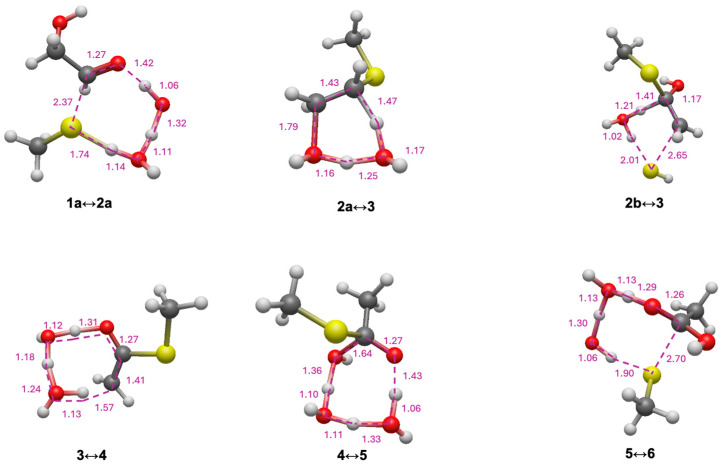
Transition state structures for C_2_ pathway (bond distances in Å).

In the formose reaction, glycolaldehyde (**1a**) is the linchpin C_2_ species regenerated in the C_4_ → C_2_ + C_2_ retro-aldol reaction. Figure 2 shows the free energy changes for conversion of glycolaldehyde to its thioester (and subsequent hydrolysis) with CH_3_SH as the reacting thiol. Relevant transition states along the pathway are shown in Figure 3. The first step is adding CH_3_SH (*G*_rel_ = −19.2 kcal) to **1a** (*G*_rel_ = −0.5 kcal) to form **2a** (*G*_rel_ = −19.2 kcal). In the potential energy curve of Figure 2, the starting point includes both CH_3_SH and **1a**, and the sum of their *G*_rel_ values is −19.7 kcal. Thus, this reaction is marginally endergonic with Δ*G* = −19.2 − (−19.7) = +0.5 kcal. Since the transition state has *G*_rel_ = −0.3 kcal, the forward reaction barrier is Δ*G*^‡^ = −0.3 − (−19.7) = 19.4 kcal. The optimal eight-center transition state (**1a↔2a**) includes two explicit solvent (water) molecules. The O…H distances for proton transfers are in the 1.05 to 1.45 Å range; the forming C…S distance is 2.37 Å and the breaking S…H distance is 1.74 Å; these are 20–40% longer than the typical C–S and S–H single covalent bonds as expected. For the remainder of this paper, we will not explicitly show the calculation of Δ*G* and Δ*G*^‡^ from *G*_rel_ values; since our Figures provide all the *G*_rel_ values. Key Δ*G* and Δ*G*^‡^ values are also explicitly shown in the potential energy curves.

Dehydration of **2a** to **3** is mildly exergonic (Δ*G* = −1.4 kcal), and is the rate-determining step (highest *G*_rel_ in the pathway) with a barrier of Δ*G*^‡^ = +26.0 kcal. The optimal six-center transition state (**2a↔3**) has one additional solvent molecule. The breaking C…O and C…H distances are 1.79 Å and 1.47 Å, respectively, in the expected range.

Enol-to-keto isomerization of **3** leads to the thioester **4**; this reaction is highly exergonic (Δ*G* = −21.6 kcal) with a low barrier (Δ*G*^‡^ = +9.4 kcal). The C…H forming bond of 1.57 Å in transition state **3↔4** is a little long but still reasonable (other distances are as expected). Comparing the thioester **4** to glycolaldehyde **1a**, we see that *intra*molecular disproportionation has taken place. **1a** has carbon oxidation numbers of +1 and +2 while **4** has carbon oxidation numbers of 0 and +3. This redox disproportionation is the thermodynamic driving force for converting a sugar into a thioester. The net change in Δ*G* from **1a** to **4** is −22.5 kcal (as indicated in Figure 2).

Hydrolysis of thioester to carboxylic acid proceeds in two steps. First, a nucleophilic addition of water to **4** forms the tetrahedral intermediate **5**, followed by elimination of CH_3_SH to form acetic acid **6**. The conversion of **4** to **6** is exergonic (Δ*G* = −6.1 kcal); this is the first of several examples where the exergonicity of thioester hydrolysis under standard conditions is not too different from ATP hydrolysis (at pH 7, *K*_eq_’ = 6.3 × 10^4^, Δ*G*’ ° = −6.5 kcal, from eQuilibrator [50]). Note that because CH_3_SH is a separate product, the energy curve in Figure 2 includes this value and the total *G*_rel_ of the combined final products is −48.2 kcal. (The same will be true for energy curves in subsequent Figures). In Figure 3, Transition state **4↔5** involving the addition of water has a shorter C…O forming bond at 1.64 Å but still reasonable. For the elimination of CH_3_SH (transition state **5↔6**), the C…S bond is noticeably longer than expected (2.70 Å) but we verified that this breaking bond is one of the eigenvectors in the transition state.

In the sulfur analog of the formose reaction [23], the C_2_ linchpin species is mercaptoaldehyde (**1b**). As shown in Figure 2, starting from **1b**, adding CH_3_SH to form **2b** is similarly mildly endergonic (Δ*G* = +0.7 kcal), while the barrier is 3 kcal lower (Δ*G*^‡^ = +16.3 kcal) compared to starting from glycolaldehyde **1a**. This is similar to what we found in our previous study; several of the formose reaction thiol analogs had barriers lowered by 2–3 kcal compared to the non-sulfur counterparts. However, forming the enol **3** from **2b** requires elimination of H_2_S which is kinetically very unfavorable with a very high barrier (Δ*G*^‡^ = +53.3 kcal). This step is also endergonic (Δ*G* = +3.6 kcal) but not hugely so. The transition state has a slightly longer C…S breaking bond (2.65 Å). The remainder of the pathway to the thioester and subsequent hydrolysis to form acetic acid is similar.

Since the transition state with the highest energies either involves the removal of water (from glycolaldehyde hemithioacetal **2a**) or removal of H_2_S (from mercaptoaldehyde hemithioacetal **2b**), a pathway that avoids this step would be kinetically more feasible. Having a carbonyl group alpha to the hemithioacetal avoids this difficult step, since only keto-enol isomerization is needed to form the thioester. The smallest molecule that can do this is glyoxal.

### 3.2. Glyoxal

While most studies of the formose reaction focus on the sugar product distribution, others have noted the presence of oxidized and reduced species formed in this complex reaction. Starting with glycolaldehyde aldol reactions, glyoxal was observed in a detailed study tracking the carbonyl migrations and epimerizations in sugars [51]. Thus, we expect glyoxal to be present in messy formose systems.

**Figure 4:**
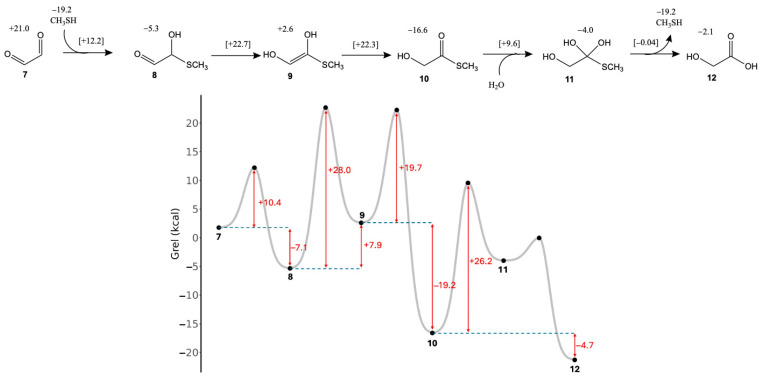
Formation of glycolic acid thioester from glyoxal (*G*_rel_ in kcal).

As shown in Figure 4, starting from glyoxal (**7**), the addition of CH_3_SH to produce hemithioacetal **8** is exergonic (Δ*G* = −7.1 kcal) with a low barrier (Δ*G*^‡^ = +10.4 kcal). This first addition reaction is more favorable compared to glycolaldehyde, because glyoxal is a higher-energy (or “activated”) species in the thermodynamic landscape [34]. Enolization of **8** to **9** is endergonic (Δ*G* = +7.9 kcal) with a moderately high barrier (Δ*G*^‡^ = +28.0 kcal); this is the rate-determining step. A second enolization to form the thioester **10** is highly exergonic (Δ*G* = −19.2 kcal) with a modest barrier (Δ*G*^‡^ = +19.7 kcal); the thermodynamic favorability is driven by the intramolecular disproportionation as expected. While there may be a possible direct route from **8** to **10** avoiding the transient enol **9**, we were not able to find a suitable transition state that involves moving four hydrogens in a single step. Once thioester **10** is formed, it can be hydrolyzed in a two-step reaction going through the tetrahedral intermediate **11** before forming the final product glycolic acid **12**. The hydrolysis is overall exergonic by 4.7 kcal. All transition states along this pathway look similar to their analogs in Figure 3 with distances of breaking and forming bonds in the expected range. (Cartesian coordinates of all transition states are provided in the Supplementary Materials.)

### 3.3. Glyceraldehyde and Its Sulfur Analogs

**Figure 5:**
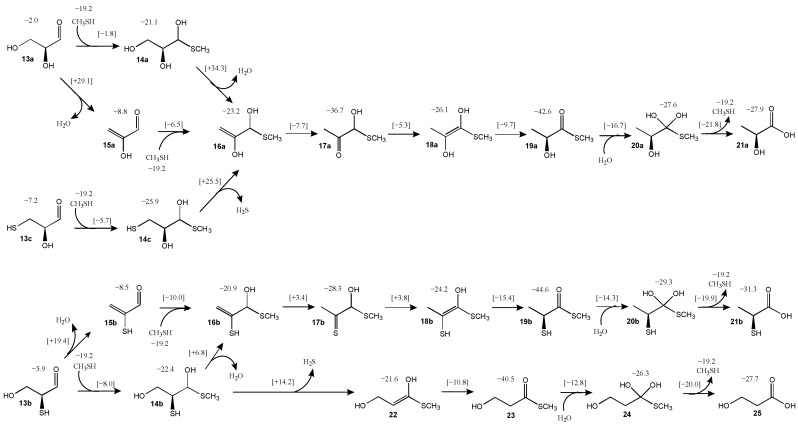
Pathways for formation of thioesters from C_3_ aldehydes (*G*_rel_ in kcal).

As discussed in the Introduction, glyceraldehyde **13a** is the sugar used in Weber’s experiments; we use CH_3_SH as the representative thiol in our computations instead of N-acetylcysteine [24]. The pathway and its energetics are shown in Figure 5. Adding CH_3_SH to glyceraldehyde to form **14a** is energetically break-even (Δ*G* = +0.1 kcal) with a barrier (Δ*G*^‡^) of 19.4 kcal; these values are similar to what we found for the addition of CH_3_SH to glycolaldehyde discussed earlier. Alternatively, glyceraldehyde could first dehydrate to the enol of methylglyoxal **15a** (the enol is favored because of the conjugated π-system); this reaction is exergonic (Δ*G* = −6.8 kcal) but it has a relatively high barrier (Δ*G*^‡^ = +31.1 kcal). Subsequent addition of CH_3_SH and **15a** to form **16a** is slightly endothermic (Δ*G* = +4.8 kcal) with a modest barrier (Δ*G*^‡^ = +21.5 kcal). The pathway **13a** → **15a** → **16a** is kinetically preferred over **13a** → **14a** → **16a** which has a prohibitively high dehydration barrier (Δ*G*^‡^ = +55.4 kcal). While **14a** → **16a** does not lead to a stabilizing conjugated π-system (compared to **13a** → **15a**), it is unclear why our calculated barrier for the **14a↔16a** transition state is so much larger even though the transition states look quite similar.

Progressing along the pathway, tautomerization of enol **16a** to the ketone **17a** is significantly exergonic (Δ*G* = −13.5 kcal) with a relatively low barrier (Δ*G*^‡^ = +15.5 kcal). Getting to the thioester stepwise requires two more tautomerization steps. Converting **17a** to the enol **18a** is endergonic (Δ*G* = +10.5 kcal). Compared to **16a**, enol **18a** is more stable by 3.0 kcal, although its transition state **17a↔18a** (*G*_rel_ = −5.3 kcal) is higher in energy than **16a↔17a** (*G*_rel_ = −7.7 kcal).

**Figure 6:**
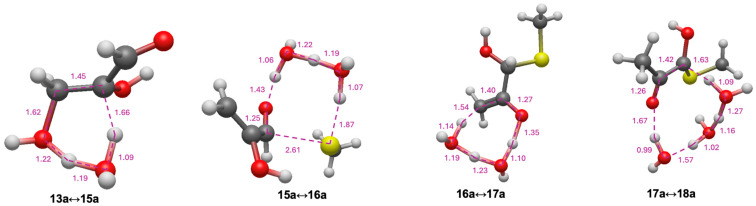
First four transition state structures for glycolaldehyde pathway (bond distances in Å).

The transition states for **13a** → **15a** → **16a** → **17a** → **18a** are shown in Figure 6. As with the C_2_ system starting with glycolaldehyde, the optimal transition state for dehydration (**13a↔15a**) is a six-center state; the breaking C…O and C…H distances are 1.62 Å and 1.66 Å, respectively, indicating that breaking the C–H bond proceeds ahead of the C–O bond. For addition of CH_3_SH, the optimal transition state **15a↔16a**) is eight-center; the forming C…S distance is 2.61 Å and the breaking S…H distance is 1.87 Å, both a little longer than the C_2_ case, but still within the expected range. The two subsequent enolization steps have transition state bond distances as expected: In **16a↔17a** the forming C…H bond is 1.54 Å; and in **17a↔18a** the breaking C…H bond is 1.63 Å.

**Figure 7:**
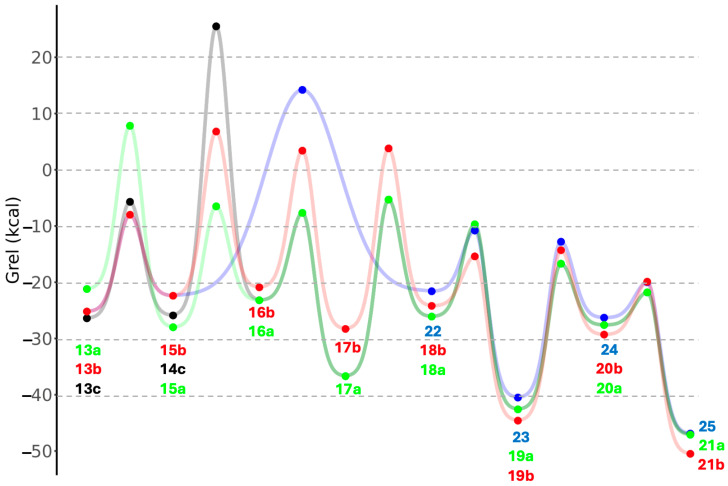
Energy diagram for C_3_ pathways superimposed (*G*_rel_ in kcal).

Tautomerization of enol **18a** to the thioester **19a** is significantly exergonic (Δ*G* = −16.5 kcal) with a relatively low barrier (Δ*G*^‡^ = +16.4 kcal). The overall exergonicity from sugar to thioester for the C_3_ (**13a** to **19a**) is 21.4 kcal, similar to its C_2_ analog (**1a** to **4**) of 22.5 kcal. We attempted to find pathways that bypass **17a** (for example a 1,3-H-shift of **16a** to **18a**) but the transition states were all higher in energy. Once the thioester **19a** is formed, it can undergo hydrolysis to lactic acid **21a** (via tetrahedral intermediate **20a**); this reaction is overall exergonic by 4.5 kcal. The transition states converting **18a** to **21a** are analogous to the ones from **3** to **6**, with no surprises in the bond distances of the breaking and forming bonds. The overall energy pathway of **13a** to **21a** can be seen in Figure 7 by following the green curve with the green dots.

We now examine the sulfur analogs. Starting from 2-thioglyceraldehyde **13b**, dehydration leads to **15b**. The reaction is exergonic (Δ*G* = −2.6 kcal) although less so than the dehydration of **13a** to **15a**. The barrier for **13b** → **15b** is 25.3 kcal, which interestingly is noticeably lower than the 33.1 kcal for **13a** → **15a**. Addition of CH_3_SH to **15b** to form **16b** is endergonic (Δ*G* = +6.8 kcal) due to the loss of the π-conjugated system, although the barrier is relatively low (Δ*G*^‡^ = +17.7 kcal). An alternative route to **16b** is to first add CH_3_SH to **13b** to form **14b** (Δ*G* = +2.7 kcal, Δ*G*^‡^ = +17.1 kcal) followed by the dehydration of **14b** to **16b** (Δ*G* = −1.5 kcal, Δ*G*^‡^ = +29.2 kcal).

Tautomerization of thioenol **16b** to the thione **17b** is exergonic (Δ*G* = −7.4 kcal) with a modest barrier (Δ*G*^‡^ = +24.3 kcal). Tautomerizing **17b** to **18b** is slightly endergonic (Δ*G* = +4.1 kcal) and the transition state **17b↔18b** (*G*_rel_ = +3.8 kcal) has a similar relative energy to **16b↔17b** (*G*_rel_ = +3.3 kcal). Tautomerization of enol **18b** to the thioester **19b** is significantly exergonic (Δ*G* = −20.4 kcal), very similar to its non-sulfur analog, and has a low barrier (Δ*G*^‡^ = +8.8 kcal). Hydrolysis of the thioester **19b** to its acid **21b** (via tetrahedral intermediate **20b**) is exergonic by 5.9 kcal, once again, not too different from ATP hydrolysis. This overall energy pathway of **13b** to **21b** is indicated by the red curve in Figure 7.

Starting from 2-thioglyceraldehyde, another possible route is to eliminate H_2_S which converts **14b** to **22**. This reaction is barely endergonic (Δ*G* = +0.8 kcal) although it has a high barrier (Δ*G*^‡^ = +36.6 kcal); thus we expect this pathway to be less feasible. However, if a small amount of enol **22** is formed, it very favorably tautomerizes into the thioester **23** (Δ*G* = −18.9 kcal, Δ*G*^‡^ = +10.8 kcal). Hydrolysis of the thioester **23** (going through tetrahedral intermediate **24**) leads to 3-hydroxypropanoic (3HP) acid **25**, an isomer of lactic acid. 3HP is a metabolite in the 3HP/4HB cycle, an analog of the rTCA cycle. The hydrolysis of **23** to **25** is exergonic by 6.4 kcal, very similar to ATP hydrolysis. This overall energy pathway of **13b** to **25** in Figure 7 begins with the red curve and branches to the blue curve.

Another possible starting analog is 3-thioglyceraldehyde **13c**. Addition of CH_3_SH leads to **14c** (Δ*G* = +0.5 kcal, Δ*G*^‡^ = +20.7 kcal) but the subsequent elimination of H_2_S to form **16a** has a prohibitively high barrier of 50.4 kcal, so we expect this pathway not to be favored (initial gray curve in Figure 7). Alternatively, **14c** could dehydrate to the thiol analog of **22** and the subsequent pathway might proceed to eventually form 3-thiopropanoic acid, the sulfur analog of 3HP. We did not pursue this pathway although we expect the energetics to be similar.

### 3.4. Intramolecular Disproportionation with a C_4_ Sugar

**Figure 8:**
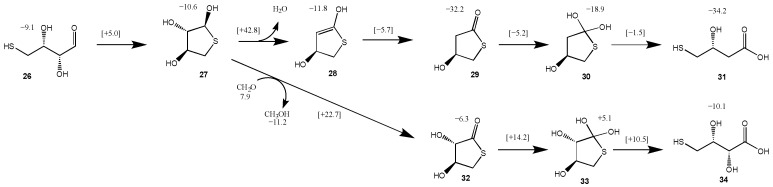
Two pathways for converting 4-thiothreose to thioesters (G_rel_ in kcal).

**Figure 9:**
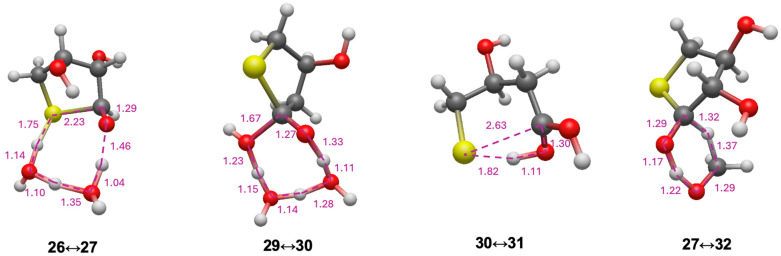
Selected transition states for converting 4-thiothreose to thioesters.

For the C_2_ and C_3_ starting sugars, the thiol (CH_3_SH) is added intermolecularly. Since we had previously explored thiol analogs of the formose reaction [23], we thought it would be interesting to consider a possible *intra*molecular addition and disproportionation starting from a 4-thioaldose. We chose 4-thiothreose **26** as our starting structure (slightly more stable than 4-thioerythrose) as a prototypical test case. The reaction pathway and energetics are shown in Figure 8. Forming the cyclic hemithioacetal **27** in an intramolecular nucleophilic addition is marginally exothermic (Δ*G* = −1.5 kcal) and kinetically very feasible with a barrier of Δ*G*^‡^ = +14.1 kcal. The transition state **26↔27** is shown in Figure 9. The ring closing C…S is 2.23 Å while the breaking S…H is 1.75 Å (with O…H distances as expected).

However, the dehydration of **27** to **28**, while marginally exothermic (Δ*G* = −1.2 kcal) has a prohibitively high barrier (Δ*G*^‡^ = +53.4 kcal), similar to our **14a↔16a** transition state. If dehydrating conditions or a suitable catalyst allowed this reaction to proceed, tautomerization of **28** to the thioester **29** is very exergonic (Δ*G* = −20.4 kcal) with a very low barrier (Δ*G*^‡^ = +6.1 kcal). Hydrolysis of **29** (going through tetrahedral intermediate **30**) leads to the acid **31**. The hydrolysis reaction is only slightly exergonic (Δ*G* = −2.0 kcal). This final product, 2-deoxy-4-thiothreonic acid, is much more stable than its reactant isomer 4-thiothreose (by 25.1 kcal). Intramolecular redox disproportionation is the driving force. The optimal **29↔30** transition-state is eight-center with all bond distances in the expected range. Interestingly, the optimal **30↔31** transition-state did not require additional explicit water molecules; the C…S breaking bond is 2.63 Å.

We explored a second disproportionation pathway since we had previously found Cannizzaro reactions to be thermodynamically favored albeit with relatively high barriers [23]. Using the reduction of CH_2_O to CH_3_OH as a coupling agent, we calculate the oxidation of **27** to form the thioester **32** to be thermodynamically favorable (Δ*G* = [(−6.3) + (−11.2)] − [(−10.6) + (+7.9)] = −14.8 kcal); the barrier is 25.4 kcal, very similar to the Cannizzaro disproportionation of formaldehyde. Hydrolysis of the thioester **32** leads to 4-thiothreonic acid **34** (via tetrahedral intermediate **33**) and is overall exergonic by 4.8 kcal. The optimal transition state is six-center, very similar to the Cannizzaro reaction transition states in our previous work [23]. Thus, depending on what else is present in the system, a sugar thiol could form different thioesters (and subsequently acids) depending on whether it undergoes inter or intra-molecular disproportionation.

## 4. Conclusions

Our calculated free energies suggest that the overall pathway converting sugars to thioesters is feasible and thermodynamically favorable. The driving force for this reaction is an intramolecular disproportionation redox reaction, oxidizing an aldehyde to a thioester while concomitantly reducing a neighboring alcohol group. The standard half-cell reduction potential for a carboxylic acid to an aldehyde is significantly more negative than the reduction of alcohol to alkyl; representative values for the reduction potentials have been tabulated by Weber [52]. The exergonicity of the internal disproportionation was also reflected in our thermodynamic map of CHO compounds [34]; sugars were 22–28 kcal less stable than their isomeric dihydroxy-acid counterparts. Since the thioester is 5–7 kcal less stable than its acid, we expect sugar to thioester conversion to be exergonic by 15–23 kcal; this is indeed what we observe in our calculations.

With regard to the kinetics, dehydration of sugar to enol was typically the rate-determining step with relatively high barriers of ~30 kcal. (For the sulfur analog, removal of H_2_S had a prohibitively high barrier of over 50 kcal, and we expect not to observe such reactions.) This suggests that dehydrating environmental conditions could facilitate the reaction, or an appropriate catalyst could accelerate thioester formation from its parent sugar. In a recent study where we utilized HCOOH and NH_3_ as simple proxy catalysts for the formose reaction [49], barriers could be lowered by 4–10 kcal compared to the baseline uncatalyzed values. The challenge for dehydrating conditions is that it would also promote other competing oligomerization reactions of sugars.

The dehydration step can be avoided if the starting compound is an alpha-keto aldehyde. Glyoxal is the smallest such example, although it is not usually considered a sugar. In this case, an enolization step is rate-determining. This is also true for the C_3_ compound methylglyoxal, which is one of two isomers of dehydrated glyceraldehyde. (The other isomer is malondialdehyde.) In this work, we have not looked at the possibility of starting with an alpha-keto acid, the smallest example being glyoxilic acid, given that it is thermodynamically a high-energy or “activated” species [34], although we are actively investigating this possibility because of recent interesting results on a glyoxylose system as a proto-metabolic precursor [53].

Our current work does not directly address if thioesters could have been precursors to phosphates in energy transduction of a protometabolic system; however, it is intriguing that our calculated free energies of thioester hydrolysis are very similar to ATP hydrolysis under standard conditions. Future work includes addressing this issue, and examining the possible compounds within this system that could act as prebiotic catalysts and facilitate thioester formation. This includes possible Cannizzaro-like reactions which have barriers in the ~30 kcal range but are also thermodynamically favorable through redox disproportionation, although this would be intermolecular rather than intramolecular.

We hope our work encourages others to revisit the “sugar system” first proposed by Weber. While Weber’s experiments only considered glyceraldehyde as the starting sugar, our work suggests that a wider range of aldehydes could be tested experimentally to examine if our proposed mechanisms are reasonable. In particular, wet-dry cycles may lead to dehydrated sugars with an alpha-carbonyl adjacent to the aldehyde facilitating thioester formation. Using thiols that are prebiotically plausible forerunners to glutathione, along with simple prebiotic catalysts, may also shed light on the metabolic origins of the glyoxalase enzyme. In future computational work, we plan to expand this system and explore if it can lead to a model of robust proto-metabolism with the inclusion of nitrogen-containing species and a wider range of prebiotically plausible compounds.

## Data Availability

The data presented in this study are available in Supplementary Materials.

## Supplementary Materials

The following supporting information can be downloaded at: https://www.mdpi.com/article/10.3390/life15081189/s1, Table S1: Energy breakdown of molecules and transition state Cartesian coordinates.
