# Supplementary material for: Sugars to Acids via Thioesters: A Computational Study
Source: Life (Basel). 2025 Jul 26;15(8):1189. doi: 10.3390/life15081189 (PMC12387730; doi:10.3390/life15081189)
Supplement: Supplementary file 1 [file life-15-01189-s001.zip › life-3701341-supplementary.pdf]

Part 1: Energy Breakdown for all structures

Table S1 contains the energy breakdown for calculated structures in this article. The computational methods section describes each of these categories: E_{elec} is the electronic energy in atomic units. All other values are in kcal/mol. E_{solv} is the solvation energy when the molecule is embedded in a dielectric. H_{corr} includes the zero-point-energy and standard enthalpy corrections to 298 K. The entropic correction ($-0.5TS_{corr}$) is half of the standard gas-phase entropy correction at 298 K. The last column (G_{298}) is the total free energy obtained by adding the other four columns (where the atomic units of E_{elec} are converted to kcal/mol by multiplying 627.5096. The transition states are labeled with a double-headed arrow between each structure.

Table S1. Energy breakdown of molecules and transition states

	E_{elec} (a.u.)	E_{solv}	H_{corr}	$-0.5TS_{corr}$	G_{298}	G_{rel}
**1a**	-229.11010	-7.26	41.58	-10.08	-143744.54	-0.50
**1b**	-552.08770	-4.32	38.78	-10.75	-346416.62	-5.71
**2a**	-667.86572	-9.50	77.48	-12.91	-419037.08	-19.22
**2b**	-990.84407	-5.62	74.32	-13.45	-621708.92	-24.21
**3**	-591.39697	-6.48	58.11	-11.68	-371067.32	-20.64
**4**	-591.43626	-3.01	57.70	-11.61	-371088.86	-42.18
**5**	-667.87962	-9.6	76.54	-12.78	-419046.71	-28.89
**6**	-229.15643	-7.20	42.15	-10.22	-143773.12	-29.09
**7**	-227.88557	-4.20	26.33	-9.86	-142988.12	21.00
**8**	-666.65250	-6.93	62.03	-12.52	-418288.27	-5.35
**9**	-666.63928	-7.42	62.22	-12.58	-418280.32	2.60
**10**	-666.66769	-8.60	62.25	-12.81	-418299.54	-16.62
**11**	-743.11749	-11.87	80.65	-13.47	-466258.05	-3.99
**12**	-304.38813	-10.88	45.93	-10.91	-190982.34	-2.08
**13a**	-343.67597	-10.51	74.18	-11.78	-215618.08	-2.02
**13b**	-666.65298	-6.92	61.41	-12.2	-418288.86	-5.94
**13c**	-666.65318	-7.43	60.96	-12.35	-418290.09	-7.17
**14a**	-782.43030	-13.86	99.87	-14.48	-490910.99	-21.12
**14b**	-1105.39915	-11.99	96.61	-15.18	-693579.13	-22.41
**14c**	-1105.40945	-9.31	96.66	-14.94	-693582.63	-25.91
**15a**	-267.21991	-5.64	45.48	-10.54	-167653.75	-8.83
**15b**	-590.18776	-2.94	42.31	-11.13	-370320.25	-8.47
**16a**	-705.96359	-10.07	80.59	-13.54	-442941.94	-23.21
**16b**	-1028.93171	-5.43	77.46	-13.96	-645606.46	-20.88
**17a**	-705.98147	-10.9	79.57	-13.86	-442955.34	-36.65
**17b**	-1028.93982	-8.68	78.45	-14.04	-645613.89	-28.31
**18a**	-705.97388	-6.45	80.64	-13.63	-442944.83	-26.14
**18b**	-1028.93950	-3.59	77.47	-14.25	-645609.78	-24.20
**19a**	-705.99823	-7.67	80.64	-13.62	-442961.318	-42.62
**19b**	-1028.97301	-3.29	77.17	-13.65	-645630.204	-44.62

**20a**	-782.44388	-10.75	98.88	-14.51	-490917.43	-27.60
**20b**	-1105.41582	-7.99	95.82	-14.77	-693585.98	-29.26
**21a**	-343.71811	-10.12	64.39	-11.79	-215643.93	-27.90
**21b**	-666.69197	-7.89	61.58	-12.31	-418314.23	-31.31
**22**	-705.96548	-7.83	81.15	-13.44	-442940.24	-21.55
**23**	-705.99766	-6.48	81.15	-13.52	-442959.15	-40.46
**24**	-782.44012	-12.16	99.23	-14.54	-490916.15	-26.32
**25**	-343.71803	-10.65	64.97	-11.63	-215643.68	-27.65
**26**	-781.21843	-11.34	83.38	-14.00	-490164.02	-9.09
**27**	-781.21349	-18.62	85.57	-13.48	-490165.49	-10.56
**28**	-704.75368	-10.59	66.90	-12.22	-442195.61	-11.82
**29**	-704.78471	-11.46	66.89	-12.22	-442215.96	-32.17
**30**	-781.23332	-14.62	85.26	-13.02	-490173.78	-18.85
**31**	-781.25484	-14.06	83.93	-14.04	-490189.08	-34.15
**32**	-780.02082	-13.14	70.46	-13.12	-489426.35	-6.32
**33**	-856.46783	-19.19	88.84	-13.89	-537386.03	5.14
**34**	-856.49652	-14.56	87.69	-14.59	-537401.25	-10.08
**1a↔2a**	-820.75435	-19.59	106.59	-16.19	-514960.42	-0.29
**2a↔3**	-668.99825	-14.83	85.15	-13.46	-419745.96	+6.80
**3↔4**	-744.30442	-14.75	87.08	-14.29	-467000.13	-11.17
**4↔5**	-820.77982	-17.83	106.13	-14.87	-514973.78	-13.65
**5↔6**	-820.78738	-19.92	106.44	-16.15	-514981.59	-21.46
**1b↔2b**	-1143.72610	-23.15	103.28	-16.63	-717635.62	-8.62
**2b↔3**	-1143.67288	-18.97	103.82	-17.02	-717597.89	+29.10
**7↔8**	-819.53926	-19.79	91.10	-15.53	-514212.98	+12.22
**8↔9**	-819.51756	-23.19	90.90	-15.07	-514202.50	+22.70
**9↔10**	-819.52466	-18.68	90.44	-15.08	-514202.92	+22.28
**10↔11**	-896.01100	-25.45	110.27	-16.10	-562186.78	+9.56
**11↔12**	-896.03300	-22.29	111.83	-16.61	-562196.38	-0.04
**13a↔14a**	-935.31873	-23.55	128.41	-17.33	-686833.95	-1.80
**13a↔15a**	-420.05995	-31.85	76.40	-13.06	-263560.16	+29.06
**14a↔16a**	-858.79078	-22.56	111.13	-15.79	-538826.68	+34.33
**15a↔16a**	-858.86301	-16.66	110.32	-16.37	-538867.49	-6.48
**16a↔17a**	-858.85265	-23.86	109.31	-15.85	-538868.68	-7.67
**17a↔18a**	-935.32889	-19.78	128.06	-17.82	-586837.40	-5.25
**18a↔19a**	-935.33951	-18.03	128.46	-17.72	-586841.81	-9.66
**19a↔20a**	-935.33866	-26.15	128.34	-17.05	-586848.84	-16.73
**20a↔21a**	-935.35719	-21.81	130.41	-16.88	-586853.89	-21.78
**13c↔14c**	-1258.29631	-19.05	125.44	-18.05	-789504.68	-5.68
**14c↔16a**	-1258.23617	-25.67	125.73	-18.26	-789473.47	+25.53
**13b↔14b**	-1258.29540	-22.84	126.42	-18.14	-789507.00	-8.00
**14b↔22**	-1258.24120	-33.58	125.56	-18.39	-789484.82	+14.18
**22↔23**	-858.85992	-22.28	109.61	-16.27	-538871.79	-10.82
**23↔24**	-935.33563	-24.33	128.66	-17.18	-586844.94	-12.83
**24↔25**	-935.34167	-27.56	129.22	-17.86	-586852.08	-19.97
**13b↔15b**	-819.51950	-27.19	92.40	-14.67	-514205.81	+19.39

**14b↔16b**	-1258.26924	-23.06	124.45	-17.53	-789492.17	+6.83
**15b↔16b**	-1181.82479	-20.82	106.43	-17.09	-741537.87	-10.01
**16b↔17b**	-1105.34018	-18.27	91.33	-14.85	-693553.37	+3.35
**17b↔18b**	-1105.33942	-17.45	90.85	-15.19	-693552.89	+3.83
**18b↔19b**	-1181.84055	-16.93	106.65	-16.68	-741543.25	-15.39
**19b↔20b**	-1258.32258	-13.95	127.44	-17.29	-789513.31	-14.31
**20b↔21b**	-1181.85793	-13.46	109.12	-16.25	-741547.80	-19.94
**26↔27**	-934.11661	-24.65	115.32	-15.72	-586092.19	+5.02
**27↔28**	-934.04585	-29.58	114.10	-16.21	-586054.42	+42.79
**28↔29**	-857.65810	-24.27	95.86	-14.65	-538131.74	-5.67
**29↔30**	-934.11867	-32.00	113.81	-15.82	-586102.44	-5.23
**30↔31**	-781.19091	-20.35	81.90	-13.21	-490156.46	-1.53
**27↔32**	-895.72261	-16.51	101.71	-14.89	-562004.23	+22.72
**32↔33**	-1009.37299	-31.12	119.40	-16.25	-633319.22	+14.23
**33↔34**	-1009.38061	-30.56	120.14	-16.56	-633322.99	+10.46

Part 2: XYZ coordinates for transition states

1a↔2a

C1	0.2536823215	-0.4758299129	0.5992088818
O2	-0.3810654960	-0.9984799864	1.5604848114
H3	-0.3023993812	0.0512510695	-0.1927251869
S9	1.2061848912	1.5677061726	1.3324581998
H10	-0.3252152992	2.2000848821	1.8716705343
O6	-1.3460889105	2.4695307993	2.3007768805
H7	-1.9011524691	1.5090580222	2.2859648660
H8	-1.2069837115	2.7521421520	3.2134946900
O9	-2.3942622214	0.2838416552	2.2127523928
H11	-3.1055800386	0.1688678579	1.5759635122
H12	-1.5859240777	-0.3383383164	1.9288557411
C12	1.5269545999	2.4806463741	-0.2262486761
H13	0.7782989694	2.2444113097	-0.9863469477
H14	1.5114177982	3.5549253638	-0.0402148334
H15	2.5129793070	2.2092567777	-0.6042978855
C16	1.5100199398	-1.1902740799	0.1343443024
H17	2.0426655691	-0.6155878891	-0.6236538748
H18	2.1687947169	-1.3549890742	0.9939591721
O19	1.1215337443	-2.4173282317	-0.4819391511
H20	0.6315360577	-2.9094900224	0.1867465058

2a↔3

C1	-0.0815887371	-0.2926435848	0.4915873199
H2	-0.3337280681	-1.7012368931	0.8096001861
H3	0.8927783834	-0.0935936570	0.9374809422
C6	-1.1948005685	0.3371046450	1.1374547862
H7	-0.9929009067	0.9886222313	1.9849245254

H8	-2.0110355618	0.6833309886	0.5144546336
O9	-2.1946602273	-0.8474541687	2.0289020448
H10	-2.0583946214	-0.6767069274	2.9683719816
S9	-0.0668847012	-0.3834803444	-1.2998782151
O10	-0.8912667533	-2.6530065180	1.2057199688
H11	-1.6769398309	-1.8458265089	1.7558259390
H12	-0.3104452874	-3.1393306902	1.8015988352
C13	0.3225211186	1.3357217228	-1.8150365352
H14	0.3914204444	1.3310435293	-2.9047910035
H15	-0.4625454487	2.0320993715	-1.5146668453
H16	1.2804963213	1.6566649521	-1.4019332060

3↔4

C1	-0.0743841904	-0.2728803435	0.0337740682
C2	-0.8599187970	-1.2277870426	0.7086284423
S3	1.2943019631	-0.8998699835	-0.9417707499
C4	2.2249787159	0.6367203054	-1.2626642443
O5	-0.2030416409	0.9882055253	0.1084136681
H6	-1.9127776248	-0.9636577006	0.8020556378
H7	-0.6795670072	-2.2709101089	0.4684396360
H8	3.0245754944	0.3675243976	-1.9538342934
H9	1.5712852243	1.3788654010	-1.7159925575
H10	2.6490282822	1.0311700027	-0.3401291798
H11	-0.7620812540	1.4288559536	1.2119488182
O12	-1.1137686661	1.6898889376	2.2417141906
H13	-0.8004826644	0.7235746176	2.8348599777
H14	-2.0689211254	1.8062509844	2.2283381043
O15	-0.3508557787	-0.3863895611	3.1733362779
H16	-0.8734867116	-0.8534390629	3.8338950761
H17	-0.4750929678	-0.9008340654	2.1800157140

4↔5

C1	0.3391845993	0.2628844845	0.8385692570
O2	0.4645157432	-0.3119410133	-0.2909882556
H3	2.3778144818	1.5096809271	0.5356604074
O4	1.8089700000	0.6768380720	1.4471111438
H5	2.2842528660	-0.1630040185	1.5036596425
H6	2.0227084159	2.8988689677	-0.2095026229
O7	2.6789378094	2.1864826558	-0.2786480463
H8	2.3698294868	1.6144948832	-1.1811302101
H9	1.1420582414	0.3082010646	-1.3905191103
O10	1.7585865954	0.8365257782	-2.0664291249
H11	2.3103725003	0.2016751732	-2.5333171986
C12	-0.2926701538	-0.5637720297	1.9494134041
H13	0.2428824440	-1.5149058694	2.0228057472
S16	-0.4606780787	1.9972167734	0.6860480816
C17	-0.4188403448	2.6596768065	2.3941445518
H18	-0.6408047199	3.7257432119	2.3288986619

H19	-1.1733508717	2.1842868095	3.0210198935
H20	0.5701876327	2.5219489324	2.8297657325
H21	-0.2707232867	-0.0643897687	2.9173589708
H22	-1.3258292411	-0.7779007154	1.6717886608

5↔6

C5	-0.7028538184	0.7389709428	1.0602275034
H6	-1.3418876350	1.3411624467	0.4186204794
C7	0.3716245133	1.5878267858	1.6707798819
S8	-1.4029930154	3.0688402830	3.0681049652
C9	-0.1265340541	3.5887199910	4.2798887177
H10	-0.5249504808	4.3504559015	4.9515474258
H11	0.1733855426	2.7286970553	4.8825624160
H12	0.7562444777	3.9999568657	3.7797499262
O15	0.9824768104	1.0058171852	2.7078148409
H16	1.7290049764	1.5712268929	2.9529719421
O17	1.0005764696	2.4833432222	1.0485902105
H18	0.3472089802	3.2156166150	0.2083408466
O19	-0.2191281003	3.9210142645	-0.4642429856
H20	-0.8686923201	4.5712667087	0.1849540763
H21	0.4068205196	4.4646050374	-0.9549377599
O22	-1.5536380555	5.2296373621	1.0720326393
H23	-1.5178624396	4.5595753496	1.8914196488
H24	-2.4886107781	5.3335804923	0.8608352063
H19	-1.2970643589	0.2575903905	1.8317699137
H22	-0.2084020639	-0.0274658578	0.4488155793

1b↔2b

C1	0.3022165476	-0.3801523096	0.0441508168
C2	1.6664994552	0.1357954260	-0.3669221441
O3	2.5540535630	0.3671236470	0.4897606486
S4	-0.5128036754	0.9669024935	1.0474364824
H5	-0.3093194861	-0.5900896671	-0.8307222881
H6	0.3911340347	-1.2736767932	0.6593644118
H7	1.6856699260	0.7079599049	-1.2990048260
H8	-1.7770544169	0.5389556796	0.8528507191
S9	2.4262061331	-1.8786660740	-1.5391501780
C10	1.0459609507	-2.7604281760	-2.3760336770
H11	2.6917812266	-2.7938030646	0.0035513372
H12	0.6451326088	-2.1335833528	-3.1741597981
H13	0.2350079314	-3.0174803001	-1.6901693741
H14	1.4244924403	-3.6788918551	-2.8280077605
O15	2.6691107872	-1.2283032769	2.3782280040
H16	2.6100881962	-0.4744320599	1.6355362222
H17	1.9300031736	-1.1134088935	2.9836865034
O18	2.8783678258	-3.1986521360	1.0119670664
H19	2.2741512825	-3.9305734437	1.1777567548
H20	2.7468767180	-2.3242300239	1.7447511989

2b↔3

C1	0.1920696535	0.1858964517	-0.2453566118
C2	1.5552933598	0.0828604152	0.0117763455
O3	-0.2105749853	-0.1318797538	-1.5510069237
S4	-0.6646175602	1.5929905607	0.5552335783
C5	-2.4035654749	1.0384175033	0.4466459520
S6	2.2208445786	-1.9995899511	1.5084820449
H7	-0.2749432179	-0.8811802914	0.5550473858
H8	2.2074443900	-0.3004019351	-0.7602074442
H9	2.0165165118	0.6195389349	0.8249056217
H10	-1.0072942250	-0.6705321041	-1.5068200936
H11	-3.0028838543	1.8374969385	0.8829987993
H12	-2.6929729282	0.9221728583	-0.5982052855
H13	-2.5857479415	0.1149268028	0.9971813493
H14	2.9494309230	-2.7443105187	0.6536557192
O15	-0.2336252969	-1.1905351959	3.6898877362
H16	-0.0087891455	-0.2568876475	3.7765082678
H17	0.6092621828	-1.6592018793	3.7773529599
O18	-0.6499888205	-1.8762937346	1.1245115038
H19	0.2747586762	-2.3159857082	1.1409866958
H20	-0.7373598649	-1.6106347909	2.0787726691

7↔8

C1	-0.2475295081	-0.1135844185	-0.1444018513
C2	1.1027804742	0.5484769080	-0.2935945865
O3	2.1437666643	-0.0533527064	-0.2354857862
O4	-1.2321316291	0.5266898994	0.3252244266
S5	-0.5309304123	-0.3442912399	-2.4902735443
O7	-1.5710912357	2.8666104194	-0.4412292523
O8	-1.0835671944	2.4899021617	-2.7687854721
H9	-0.1851734453	-1.1966358152	0.0140793387
H10	1.0596719592	1.6526538224	-0.4185264557
H15	-2.4402903067	3.1690764181	-0.1605665078
H16	-1.4050780609	1.9066975471	-0.0327470533
H17	-1.7823584474	2.6409838179	-3.4158192709
H18	-1.3866891184	2.7537616863	-1.7023980396
C16	-2.2702099081	-0.8610172006	-2.2333694241
H19	-2.3177886036	-1.9454821352	-2.1344280063
H20	-2.6522508466	-0.3988432756	-1.3182982476
H21	-2.8847653335	-0.5599737296	-3.0826526979
H22	-0.8500777893	1.4115804829	-2.7452972796

8↔9

C1	-0.1597896912	0.0224668212	-0.2972080458
C2	0.6906082205	-0.2831087241	0.9041898266
O3	-0.4939116769	1.2523803047	-0.5536660384
O4	1.2986485262	-1.3178888565	1.0407802511

O5	-1.6969737280	1.8209359709	1.5157708252
S7	-2.0700761189	-0.9477058596	0.5391953745
C8	-1.6809491270	-2.2118659486	1.8076755418
H9	-0.0081553934	-0.6447054682	-1.1466737632
H10	0.7129852278	0.5264895751	1.6625265464
H11	-1.0077857885	1.7406156557	0.4761257955
H15	-2.0754070285	0.6490791003	1.3699188875
H16	-2.2312332070	-3.1182113263	1.5591601129
H17	-0.6110773098	-2.4293265277	1.7971126339
H18	-1.9705571379	-1.8633939490	2.7983910900
H19	-2.4639947020	2.3852574796	1.3557865137

9↔10

C1	0.0349028802	0.3361307673	-0.0484959188
C2	-0.7505611640	-0.9399984168	-0.1157564549
S3	1.6787022583	0.5659533505	0.5279342316
C4	2.0970423162	-0.8250369437	1.6459799361
O5	-0.2018816809	-2.0567984793	0.5546413738
O6	-0.4373887284	1.4239850827	-0.5809719753
H7	-1.7853103746	-0.7285327959	0.1803619813
H8	3.0294582252	-0.5085359140	2.1159783065
H9	1.3305316553	-0.9579874794	2.4037247389
H10	2.2406468817	-1.7503034240	1.0986386899
H11	-0.2896492551	-2.8105825577	-0.0344961588
H12	-1.4197263689	1.2796051203	-1.3405192831
O13	-1.2793496029	-1.1643146978	-2.6875926918
H14	-0.5098798651	-1.0705623388	-3.2612255991
H15	-0.9081762103	-1.1238005554	-1.4946597659
O16	-2.2995680191	1.0332411258	-2.1934573292
H17	-2.2693988184	1.7116660775	-2.8754726920
H18	-1.8615463064	-0.0609019309	-2.6099575096

10↔11

C1	0.2471321106	-0.2305850498	1.0037826542
C2	0.7413459847	-0.7270983612	-0.3352813624
O3	1.7519027076	0.1331250715	-0.8121352339
O4	-0.7901018826	-0.6583691764	1.5520540208
S5	-0.5436662558	2.1482357603	-0.3221371456
C6	-1.8719245467	1.5654047347	-1.4572829371
H7	1.1982014842	-1.7101207839	-0.1692834284
H8	-0.1055141158	-0.8442872062	-1.0133335403
H9	1.2736720679	0.9742754581	-1.0064760069
H10	-2.6134106527	2.3536405279	-1.5949282964
H11	-1.4575785679	1.3161677785	-2.4356948799
H12	-2.3780781907	0.6856791326	-1.0507772408
O13	-1.2143420423	0.7383561208	3.4661601979
H14	-1.6569597774	1.5813022117	3.0359591906
H15	-1.0373211024	-0.0192907960	2.5652064007

O16	1.1567458015	0.4045107040	1.7406743855
H17	-0.3246931050	1.0021368757	3.7361795718
H18	1.8704926449	0.6498289025	1.1156465524
O19	-2.1876845287	2.6588682586	2.1985745020
H20	-2.1223871388	3.5809852384	2.4664538662
H21	-1.6878790067	2.5786170985	1.3214894514

11↔12

O1	-0.4478860384	-1.1004375201	0.0550634717
C2	-0.2951553436	-0.0194012972	0.7933871102
S3	2.0502265469	0.8273139821	-0.7903748963
C4	-0.9482840397	1.2396358161	0.2680463796
O5	0.0961228668	-0.1080640063	1.9774041323
C6	2.0772041250	-0.5591492953	-2.0092031734
O7	-0.9930258263	1.2037341666	-1.1384904171
H8	-0.7381010835	-0.7735821943	-0.8229777757
H9	-1.9826709663	1.2442237884	0.6343795681
H10	-0.4248118601	2.1060504332	0.6728481906
H11	0.8160277381	-1.0213187612	2.2766322579
H12	3.1066314662	-0.8237375783	-2.2622493879
H13	1.5843158839	-1.4511468637	-1.6096316242
H14	1.5761617218	-0.2735887819	-2.9370768999
H15	-0.0483756799	1.3335386931	-1.3985334859
O16	1.6813371279	-1.8006289186	2.5888932864
H17	1.9530025126	-1.6339948114	3.5014033738
H18	2.4935430955	-1.5711258242	1.9763125261
O19	3.5533794811	-1.0281201123	1.1255346502
H20	4.0100477375	-1.6200148435	0.5184260858
H21	3.1455562952	-0.2992427434	0.5519683526

13a↔14a

C7	-2.4067763304	0.2198285073	1.2539448425
O2	-2.6548703884	1.0802937449	0.3364814887
H3	-1.9517538424	0.8665182930	-0.8508176972
S4	-3.4075226753	-1.7121312531	0.7241500570
C5	-4.9911705056	-0.9437539831	0.2313994891
H6	-5.4317389924	-1.4959394617	-0.5980972490
H7	-5.6823590020	-0.9560350332	1.0738210525
H8	-4.7911598234	0.0873796114	-0.0728835317
O9	-2.2109558884	-1.7299311231	-1.8813243902
H10	-1.4727884268	-2.3511688822	-1.8843921835
H11	-2.7190703218	-1.7794113819	-0.8262700741
O12	-1.4779191272	0.5784712388	-1.7697817411
H13	-1.8191027212	1.1538465690	-2.4609205333
H14	-1.8349266028	-0.6880602983	-1.9243409713
C24	-2.9627286854	0.5375437888	2.6377601981
H25	-4.0595902493	0.5868255291	2.5910447774
C26	-2.4586660933	1.9153332419	3.0717573245

H18	-1.3607751619	1.9218044981	3.0531285703
H19	-2.8382095867	2.6789758006	2.3868295469
O20	-2.5307589507	-0.4436578276	3.5607020169
H21	-2.6849921679	-0.0604027238	4.4332661600
H22	-1.4103558040	-0.2443813468	1.3093436721
O23	-2.9435490386	2.1058294302	4.4065075300
H24	-2.4898419962	2.8599558964	4.7919799758

13a↔15a

C1	-0.2635594434	0.0014489077	0.5292371853
H2	0.1152877848	-1.6021638754	0.7547885846
C3	1.0377618999	0.5006388835	0.8474222271
O4	1.8494566110	0.8486136335	-0.0111344260
H5	1.2955374288	0.5206429714	1.9305799267
C6	-1.3366294754	0.1099939457	1.4963011219
H7	-0.9916269447	0.3987892822	2.4883426859
H8	-2.1885156210	0.6933082964	1.1537140393
O9	-2.0447185282	-1.3275114751	1.7483022509
H10	-2.4477056850	-1.3447512332	2.6255562659
O11	-0.6469061414	0.1147800238	-0.8157042260
H12	0.1465070918	0.4748285448	-1.2469159626
O13	-0.1713958207	-2.6014437544	1.0725762070
H14	-1.1757126317	-2.1533563467	1.5354514109
H15	0.4664349272	-2.9002916206	1.7344850385

14a↔16a

C1	-0.1362248189	-0.0876814166	0.5027243791
H2	-0.1536812270	-1.5731803601	0.6262129704
C6	-1.1901718579	0.2677787954	1.4090435346
H7	-0.9389804611	0.8029999748	2.3186370555
H8	-2.1208031908	0.5758348836	0.9482275897
O9	-1.8701667023	-1.1724121967	2.2221398070
H10	-1.5789784524	-1.1083864443	3.1404861658
O11	-0.4311326962	0.1042397857	-0.8830371801
H12	-0.3834510742	1.0532225806	-1.0526068423
O13	-0.4796331897	-2.6362505389	0.9464107510
H14	-1.2886955435	-2.0115002414	1.7441376674
H15	0.2786472926	-3.0447900863	1.3803783207
C13	1.2492644340	0.3790404511	0.8477831507
H16	1.9527143540	-0.0014775864	0.1024211554
O15	1.3060429193	1.8080835058	0.8650841267
H17	2.2093273399	2.0847792690	0.6779923262
S17	1.7225945998	-0.3719577901	2.4930210897
C18	3.2130711930	0.6001399449	2.8904293815
H19	3.5490553738	0.2749915802	3.8748564686
H20	4.0122534883	0.4171826242	2.1690723334
H21	2.9683794443	1.6612395946	2.9333249869

15a↔16a

C1	0.1869173563	1.2269809729	0.1360760032
C5	-0.7893281256	0.8440295001	0.9671116700
C3	-2.2239369841	1.0777002149	0.6447948973
H4	-2.8343110891	1.3793442788	1.5048293556
O5	-2.6593795227	1.3126526078	-0.5042401194
H6	-2.1519408778	0.5799116277	-1.6172148600
S7	-2.9304210295	-1.3471121861	1.2896048108
C8	-4.7072920075	-0.9064568613	1.1697088480
H9	-5.2909806866	-1.7901641514	0.9116654968
H10	-5.0565524135	-0.5283197624	2.1310167103
H11	-4.8555775817	-0.1397570701	0.4055297148
O12	-2.1134071956	-2.2474303744	-1.3790928700
H13	-1.2750680101	-2.7086138844	-1.2527687755
H14	-2.4764341534	-1.9957616335	-0.4071162719
O15	-1.9087463507	-0.0831075883	-2.4043391586
H16	-2.5593461227	0.0446447129	-3.1035606127
H17	-1.9760918348	-1.2084659702	-1.9369948134
H18	-0.0544563291	1.7042148113	-0.8021733241
H19	1.2255775188	1.1068881999	0.4146157022
O20	-0.5290753542	0.3570109782	2.2084080810
H21	-1.2899982274	-0.1984937894	2.4601482473

16a↔17a

C1	1.0458184465	-0.2025119784	-0.3297307919
C2	-0.2014386959	-0.1268111457	0.2998388213
H3	-0.4610172437	-0.9167629211	0.9914914023
H4	-0.5290195740	0.8725167704	0.5815281177
C5	1.8764918385	-1.4810416422	-0.2356172251
H6	2.1140058950	-1.8120688603	-1.2526766113
O7	1.1929413273	-2.4801229492	0.4787299707
H8	1.5729949035	-3.3311844830	0.2446019975
S9	3.4970196789	-0.9834902572	0.5621565027
C10	4.1534807972	-2.6500539226	0.9048213894
H11	5.1187196124	-2.5043919217	1.3905307074
H12	3.5015962216	-3.1957196647	1.5877968610
H13	4.3140923977	-3.2209432740	-0.0138676661
H14	0.6961365782	1.5571894170	-1.6341568974
O15	1.5679390572	0.6917532607	-1.0716133251
O16	-1.7143684336	0.2948815599	-1.8224307160
H17	-1.1366067721	-0.0909340924	-0.9184948324
H18	-1.6963191016	-0.3710244345	-2.5201698105
O19	-0.1188606172	2.0583060338	-2.1714935655
H20	-1.0104256208	1.2149467538	-2.0829840932
H21	-0.3435635871	2.8809591269	-1.7257608873

17a↔18a

C4	0.6518784502	0.8301817772	-0.7970927364

H5	1.2108140311	-0.2621472783	-1.8637880377
C6	-0.2122339301	1.4295343071	-1.7554444371
O7	0.1974467480	2.2842131237	-2.5821372198
S5	2.1549251657	1.8128438626	-0.4293569857
O6	1.7279856074	-0.7861236023	-2.6724217947
H7	2.4230822910	0.1498029621	-3.1688330951
H8	2.2184610514	-1.5311502537	-2.3088528997
O9	1.1807713226	2.2664676804	-4.9875388537
H10	1.3332302932	3.1720361328	-5.2750874394
H11	0.5681401158	2.3295447628	-4.2097881297
O12	2.9304814398	1.1429349448	-3.4723530891
H13	2.3329313936	1.6048028432	-4.1580378889
H14	2.8353353947	1.6637499064	-2.6380732994
O15	0.1275822233	0.1370249889	0.3079692410
H16	-0.2268306794	0.7796730122	0.9379831278
C17	3.1798602389	0.5038728072	0.3364301712
H18	4.0046728185	0.9947509900	0.8520917070
H19	2.5724555238	-0.0425093988	1.0563971370
H20	3.5789342856	-0.1863561855	-0.4094163031
C21	-1.6407428289	0.9289001548	-1.8537297307
H22	-1.8319288208	0.5929451730	-2.8762945372
H23	-1.8543626907	0.1226215254	-1.1542463299
H24	-2.3151643127	1.7687653729	-1.6590437843

18a↔19a

C1	0.5231942394	0.1360556050	0.3575246415
H2	0.4409042516	0.8698163493	1.1694377358
H3	-0.3810368904	-0.4773874043	0.3637207578
C4	0.7161040638	0.7946989724	-0.9867988683
H5	0.9854053289	-0.4105538899	-2.0284021785
C6	-0.3182500377	1.4648061968	-1.6782116285
O7	-0.1101210304	2.4191479282	-2.4673489972
S8	-2.0142048352	0.8630531310	-1.4166916887
O13	1.4380133828	-1.0796418067	-2.7707612554
H14	2.3820169280	-0.2728718037	-3.1334141884
H15	1.7291904520	-1.8836244493	-2.3271484242
O16	1.5063506444	2.2174218751	-4.5966404413
H17	1.7529075715	3.0989696687	-4.8905981747
H18	0.8162668493	2.3398553236	-3.8981659468
O19	3.0479618675	0.6425535496	-3.2504853615
H20	2.5726140759	1.2525844792	-3.9022150600
H21	2.8621378334	1.0702917718	-2.3595814854
C20	-2.8672229555	1.7891509505	-2.7364968571
H19	-3.9111548984	1.8772648134	-2.4385114031
H22	-2.4107482692	2.7730632104	-2.8188350205
H23	-2.7953540832	1.2658335221	-3.6894208509
O24	1.9609761758	1.5503272182	-1.0423745168
H25	1.6944794442	2.4624177922	-1.2405941764

H24	1.3671229889	-0.5193913348	0.5925916701

19a↔20a

C1	-0.6432502154	-1.1339510003	-0.9060163692
C2	-1.2347368629	-0.2878883999	0.2108676226
C3	-1.3213166317	1.2002996703	-0.2065786240
S4	0.3768721673	2.0222299352	0.1767179273
C5	0.3426135756	3.3474765655	-1.0704827466
O6	-1.8687461776	1.4837670756	-1.3068621097
O7	-2.5242866286	-0.7936375130	0.4982843648
H8	0.3753381344	-0.8211549481	-1.1416453317
H9	-0.6311316197	-2.1801137305	-0.5939442517
H10	-1.2657842888	-1.0364224798	-1.7962180576
H11	-0.5908675569	-0.3450319880	1.1035962104
H12	1.3650988310	3.7035964615	-1.1920405829
H13	-0.0120367941	2.9285438274	-2.0124266565
H14	-0.2907710560	4.1820586314	-0.7629956404
H15	-2.9494427992	-0.1179111607	1.0418077857
O16	-3.1379813907	3.5045001400	-1.8476554392
H17	-2.5375987708	2.6346852131	-1.6887146556
H18	-2.5865584334	4.1976541588	-2.2229615913
O19	-3.8132711782	3.6096255954	0.4414578281
H20	-3.5400411796	3.7015387136	-0.6946085690
H21	-3.6487403666	4.4178340841	0.9372385661
O22	-2.2771642651	1.8102497958	1.0728673532
H23	-3.1429602027	2.8100942831	0.7854603815
H24	-1.6263301983	2.0892168415	1.7295195991

20a↔21a

C1	0.7846796492	1.9826568205	0.4162342822
C2	0.5373501444	0.4811333248	0.5553883516
C3	1.4411115210	-0.2867460103	-0.4219702636
S4	0.5445150995	-2.6882989119	0.5784087109
C5	1.8144312052	-3.4235720905	-0.5374788811
O6	0.9780273930	-0.4576405646	-1.6666637442
O7	2.6800626218	-0.3423155522	-0.2853385699
O8	0.7919485521	0.1039163472	1.8997180284
H9	0.5783634931	2.3132197612	-0.6038822306
H10	0.1278594153	2.5195302060	1.1023582083
H11	1.8209129198	2.2290272653	0.6562348270
H12	-0.5037257583	0.2522608633	0.3019285799
H13	1.8449064288	-4.5038589858	-0.3890020659
H14	1.5584968258	-3.2225793796	-1.5781136624
H15	2.8050943588	-3.0054845821	-0.3455557481
H16	0.0277812200	-0.6298440559	-1.6236796893
H17	3.1515454182	-0.3260643338	0.9234094828
H18	0.6692815227	-0.8712848576	1.8920475320
O19	2.8309967308	-2.7225089210	2.7041107246

H20	2.0100708490	-2.8694713517	2.1502500290
H21	3.3541838788	-3.5279895220	2.6505316114
O22	3.4397913552	-0.3512904221	2.0165378185
H23	3.3580480290	-1.3289698186	2.3130164083
H24	2.6344548617	0.0691581836	2.3903318914

13c↔14c

C1	-0.4155876119	1.1071662544	0.2637302136
C2	0.9768307711	0.5168258673	0.0520278671
C3	2.1023864266	1.5084960048	0.3294870425
O4	1.1975516521	-0.6616971726	0.8020607504
O5	2.0273447038	2.6415296342	-0.2321985673
S6	-1.7591210410	-0.0915615954	-0.1326787163
H7	-0.5545566385	1.3496470077	1.3174315468
H8	-0.5381286788	2.0147524013	-0.3240468725
H9	1.0947314437	0.2991654893	-1.0252917558
H10	3.0738125609	1.0137734543	0.4649285608
H11	0.4475017731	-1.2447576742	0.6249006546
H12	-1.5835246406	-0.1024830358	-1.4715108010
S13	1.9444301311	1.9194281368	2.6460909593
C14	2.5015384790	0.4119582661	3.5336886533
H15	3.3338454269	2.9881424569	2.6804754429
H16	1.8635619057	-0.4128114727	3.2243271980
H17	3.5380028623	0.1682047829	3.2972685654
H18	2.4041261268	0.5722518734	4.6082500686
O19	3.9276975865	4.1607244690	0.1955012093
H20	3.6697806120	5.0416475986	-0.0921335041
H21	3.1300220653	3.5006386272	-0.0579092238
O22	4.1581288249	3.7552379713	2.5694492303
H23	3.9175080974	4.5025511234	3.1303969586
H24	4.1039289344	4.0343097737	1.4794998442

14c↔16a

C1	-0.7225328005	-1.0827620146	-0.7160694507
C2	-0.2328530390	0.1451652373	-0.2956376012
C3	1.2664446383	0.3426059726	-0.0650811830
O4	1.7624543204	-0.4550279889	1.0194468785
O5	-0.9139511609	1.2852178571	-0.7743136590
S6	-1.8824708747	-2.6874321814	0.9780736216
S7	2.2196142300	0.0423339309	-1.6114296434
C8	3.8766027742	0.5991195794	-1.0708254017
H9	-1.6470220319	-1.1110148933	-1.2730307486
H10	-0.0801335265	-1.9430350380	-0.8139854560
H11	-0.5651432316	0.1217969152	1.1319546969
H12	1.4404203522	1.3750694010	0.2430072055
H13	1.8029629743	-1.3724329138	0.7194220671
H14	-0.9863226177	1.9276610894	-0.0600743681
H15	-3.0859754715	-2.5864906272	0.3768847803

H16	4.5820304521	0.2921205252	-1.8430659745
H17	3.9097691063	1.6846539638	-0.9679680855
H18	4.1440893537	0.1221078152	-0.1289307883
O19	-2.2060807403	-1.0224316579	3.4992195331
H20	-2.2702054118	-1.7228276028	2.7767500358
H21	-3.0967782669	-0.6793600416	3.6306517078
O22	-0.5440215079	0.3620608881	2.2749805385
H23	0.3408595782	0.0190846855	2.4926992896
H24	-1.2698703477	-0.1640092238	2.8260872434

13b↔14b

C1	-0.5375689138	0.0785954591	-0.6880305174
C2	-1.1595574291	1.3500481494	-0.1926725333
C3	0.2646920542	0.1748312386	-2.0000298442
S4	0.5224728832	-0.5017889659	0.7206676544
O5	-2.3734434540	1.3838063274	0.0795113563
O6	1.3228543460	1.0980755663	-1.9348649941
H7	-1.3305911047	-0.6631794290	-0.7959804268
H8	-0.4948847171	2.1656677812	0.0903846236
H9	0.6851940295	-0.8140021800	-2.2012868597
H10	-0.4348989118	0.4284836215	-2.8045138981
H11	1.5986571309	0.2152745996	0.3257903830
H12	0.9352238882	1.9576426161	-2.2004426111
H13	-2.4757280689	4.2344775063	-1.9547878701
S17	-0.7135757573	3.4931507197	-2.3125629871
C18	0.1701383143	4.5889782197	-1.1160193328
H16	1.0498866082	4.0863124552	-0.7063353302
H17	0.5119163428	5.4951432420	-1.6192905978
H18	-0.4738438742	4.8815746096	-0.2811287176
O19	-3.3386723384	4.6325542232	-1.5307716778
H20	-3.2377321268	5.5901765706	-1.5666225985
H21	-3.3875520135	4.0971862653	-0.3272799425
O22	-3.3474265740	3.5353714536	0.6325761834
H23	-2.8830419637	2.5447835434	0.4315455227
H24	-4.2346194572	3.4056372327	0.9860059493

14b↔22

C1	0.1131531210	0.2144733778	-0.3534218816
C2	1.5046182991	0.2278396946	-0.1947201546
O3	-0.3314116083	-0.1049639395	-1.6466255630
S4	-0.8354048019	1.5385983409	0.4892187999
C5	-2.5244867645	0.8609913402	0.3302225469
C6	2.1768597307	0.9560175919	0.9205634298
O7	3.5408378084	0.5869286620	1.0080556341
S8	2.2456790030	-2.3366450964	0.3675114358
H9	-0.2176183789	-0.8466178801	0.4783195698
H10	2.1159662838	-0.0161870310	-1.0516603347
H11	-0.9420902679	-0.8469887492	-1.5824389096

H12	-3.1925001336	1.6018977865	0.7690986808
H13	-2.7712223638	0.7417600288	-0.7241792289
H14	-2.6378184392	-0.0864839217	0.8574620867
H15	2.0795364988	2.0306367752	0.6809005095
H16	1.6382421154	0.7979563584	1.8648427010
H17	3.9761376908	1.2081335337	1.5990073391
H18	2.8835725505	-1.9503122620	1.4904325798
O19	-0.4565474269	-1.9011712483	1.0517276765
H20	-0.5935108535	-1.7154309200	1.9902248153
H21	0.6161417293	-2.2759222149	0.9183980603

22↔23

C1	0.1339216310	-0.0603559978	0.3776465962
C2	-0.6969847334	0.9460073743	-0.1613660844
C3	1.6275827927	0.1360300730	0.3627359291
O4	2.1372442250	-0.1935476158	-0.9371957926
O5	-0.5342341557	2.2012923144	-0.0490703261
S6	-2.1843707353	0.3845532521	-0.9911770927
C7	-3.1005493881	1.9533732525	-1.1635401293
H8	-0.1685785702	-1.0766920949	0.1328586158
H9	1.8709580719	1.1783368209	0.6126558639
H10	2.0933269485	-0.5060754180	1.1231099779
H11	3.0989034688	-0.1705326448	-0.8898160432
H12	-0.0336625329	2.6161090243	1.0874310998
H13	-4.0066160087	1.7144265580	-1.7207600307
H14	-3.3629068674	2.3609146159	-0.1885638273
H15	-2.5022641032	2.6751384434	-1.7144513170
O16	0.2425188639	2.8428601935	2.1485251371
H17	1.1587203829	3.1312091520	2.2086761184
H18	0.0789486115	1.8043327192	2.6617978681
O19	-0.1148726840	0.6040535695	2.9472404323
H20	-0.9769085297	0.4439859628	3.3478950822
H21	-0.1236118815	0.1915812497	1.9034740810

23↔24

C1	-0.3410427916	1.0288955296	-0.4097760372
C2	0.0305593663	-0.3704931355	0.0544893480
C3	1.5159194457	-0.4955510101	0.4092394626
S4	1.8657185883	0.2949360669	2.1225736291
C5	3.2294304295	1.4299340966	1.6906927111
O6	2.3755115710	-0.1889181238	-0.4946708082
O7	-1.6798538545	0.9653003207	-0.9079364780
H8	-0.2614428604	1.7278690259	0.4311547028
H9	0.3577165339	1.3530234049	-1.1913187115
H10	-0.5947627351	-0.6614244237	0.8999368299
H11	-0.1788208169	-1.0642827524	-0.7687483778
H12	2.8828451145	2.4631041870	1.6734876083
H13	4.0269107201	1.3193824615	2.4248327805

H14	3.5854730709	1.1348075820	0.7026341838
H15	-1.9670824818	1.8632399485	-1.0945846222
O16	2.1709836057	-1.4453794920	-2.5961877513
H17	2.2700947860	-0.8049490562	-1.7391998135
H18	1.4976704040	-1.0757945405	-3.1756711182
O19	1.6053752544	-2.0511299099	0.8040395300
H20	2.4017084270	-2.1152313983	1.3486213195
H21	1.6368750022	-2.8140045433	-0.2557301452
O22	1.6566819841	-3.3631427010	-1.2396679618
H23	0.8143349185	-3.8039259699	-1.3924011398
H24	1.8573751859	-2.5375796057	-1.9999627146

24↔25

C1	-0.7215435144	1.1555526910	0.2103634482
C2	0.0105957157	-0.1300405087	-0.1915206549
C3	1.4801953565	-0.1705977178	0.1213165973
S4	2.1207072332	1.7139115844	-1.8134008405
C5	3.7738638357	1.8923344426	-1.0330936559
O6	1.8326662043	0.5360391415	1.1950781880
O7	2.2563889943	-1.0877718835	-0.2531725136
O8	-2.1010447320	1.0762825607	-0.1380302959
H9	-0.6861071899	1.2805684253	1.2930478446
H10	-0.2294322936	2.0152783806	-0.2514223126
H11	-0.4365968207	-0.9758602985	0.3504075390
H12	-0.1170358433	-0.3316905525	-1.2555193067
H13	4.4562329303	2.4348195933	-1.6898852331
H14	3.6835281452	2.4624663314	-0.1053878984
H15	4.2173263911	0.9157619524	-0.8078825625
H16	2.7699284020	0.3595068206	1.3639801376
H17	2.0433190524	-1.6273063558	-1.3879718649
H18	-2.1745713535	1.2467729192	-1.0818015662
O19	2.6247136889	-0.2959463523	-3.9385822965
H20	3.5432729058	-0.2489473130	-4.2249790119
H21	2.4830910576	0.5051604025	-3.2757379051
O22	1.9047461952	-2.0572013128	-2.4313283819
H23	2.3348694701	-2.9136893349	-2.5215578747
H24	2.2782872326	-1.3286853913	-3.1724993513

13b↔15b

C1	-0.1991520216	0.2864233060	-1.1276780458
C2	1.1074612749	0.3186121281	-0.4415255882
C3	2.3202336868	0.1437502188	-1.1687113641
O4	3.4069088694	-0.2176318291	-0.7125641484
S5	1.1303025612	-0.4288866892	1.2288566306
O6	-1.1060917870	1.3477853129	-0.5976228376
H7	-0.0608110199	0.4790914023	-2.1966216887
H8	-0.7345650497	-0.6631871262	-0.9979371806
H9	1.5528207337	2.0570627829	-0.1812772108

H10	2.2345103104	0.4238434345	-2.2442426553
H11	2.4854665428	-0.5491898360	1.1825697625
H12	-2.0103037176	1.1364515323	-0.8569751241
O13	1.5761672710	3.0049315084	0.2406714056
H14	0.5951339407	2.7893649960	1.0749728793
H15	1.3814791047	3.6351155396	-0.4627228863
O16	-0.2384084518	2.2925045013	1.6568313028
H17	-0.7722109452	1.9242941525	0.8802755726
H18	0.2204962748	1.4779309101	1.9969118792

14b↔16b

C1	1.2970897208	-0.4130619056	-1.4774644278
C2	0.3116008406	-0.6522218748	-0.4308169168
S3	0.3067358527	-2.3319969094	0.2111528954
C4	-1.0496750657	-0.0413633423	-0.7364722988
O5	-0.9109832764	1.1781737612	-1.4450547220
S6	-2.0308228255	0.2081992729	0.8435025406
C7	-3.5169069106	0.9774141850	0.1076866226
H8	2.0997348467	-1.1459932408	-1.4951853860
H9	0.9213741202	-0.1849880750	-2.4781327470
H10	-0.2003076048	-3.0288426032	-0.8453415734
H11	-1.6821051671	-0.6666949183	-1.3794447460
H12	-0.2565192435	1.7056955702	-0.9678554912
H13	-3.2197324840	1.7793884904	-0.5675498712
H14	-4.1120976901	1.3860952282	0.9248648464
H15	-4.1117650529	0.2366003269	-0.4298899612
O16	2.0711755514	0.9991863075	-1.2006273932
H17	2.7258639084	1.0290219374	-0.2114802985
H18	2.5652538867	1.2672138697	-1.9881137667
O19	3.2434469264	1.1127132822	0.8910528905
H20	2.3738619672	0.9580380599	1.4906410016
H21	3.8364096984	0.3740236770	1.0693809061
O22	1.1346252092	0.4911461543	1.9346139651
H23	0.4181157446	1.0544804103	2.2485123463
H24	0.7903136890	0.0643151728	1.0369823896

15b↔16b

O1	-0.2720609993	1.6056841649	0.0381745256
C2	0.0486075643	0.4197824769	0.3320687102
C3	1.1614427163	-0.2708425262	-0.3703893134
C4	1.1801863812	-1.6093886499	-0.4451006217
S5	2.3933949105	0.7421997385	-1.1461624358
H6	-0.7073877329	-0.2743354719	0.7193867549
H7	1.9452060364	-2.1498479750	-0.9882633310
H8	0.4069816773	-2.1951070388	0.0384771980
H9	1.7232970752	1.8909635289	-0.9113265368
S10	1.0461409925	0.6033981395	2.5671259130
H11	-0.4941542463	1.1555943186	3.3519897697

C12	1.3933411417	-1.1135873192	3.0964090307
H13	1.7743390774	-1.6701561897	2.2348634744
H14	2.1681410408	-1.1061921226	3.8640972784
H15	0.5113482208	-1.6249314547	3.4873194359
O16	-2.0722727545	2.4909647126	1.4682909932
H17	-2.9403792756	2.3076756561	1.0979692103
H18	-1.3190319159	2.1376674514	0.7961964801
O19	-1.4529973342	1.6174654027	3.6232861561
H20	-1.8448465438	2.0443368475	2.6184038595
H21	-2.0272542551	0.9316407573	3.9829671229

16b↔17b

C1	1.2811021117	-0.9585946390	1.1042228070
C2	0.4176816832	-0.3735189415	0.1974915455
C3	-0.7471742884	-1.1712881099	-0.3505537668
S4	-2.2963115220	-0.7113224973	0.5973896478
C5	-2.9620940917	0.6530553081	-0.4186749189
O6	-0.5117290550	-2.5461050731	-0.1865958151
S7	0.6613095753	1.2091420753	-0.4814800324
H8	1.0791025650	-1.9678652363	1.4404590651
H9	1.8211155431	-0.3099432502	1.7891186832
H10	-0.9272133963	-0.8990130626	-1.3945322102
H11	-3.9224108821	0.9167085627	0.0263751419
H12	-3.1207708952	0.3349286964	-1.4499716299
H13	-2.2963992976	1.5138319982	-0.3885084354
H14	-1.3290397870	-3.0096549282	-0.4015691529
H15	2.4269316927	0.5901303344	-0.6817727440
H16	3.1856528289	-0.1680974536	-0.5593719740
H17	2.4955044706	-0.7726230168	0.1006679223
H18	3.8757522041	0.2009009877	0.0090236880

17b↔18b

S1	1.8871120584	-1.0336456603	0.6686569402
C2	1.1612057446	0.4427015722	0.1455197075
C3	2.0778929905	1.5893154734	-0.2005365777
C4	-0.2107149652	0.5934637999	-0.1210987952
O5	-0.6301333935	1.7788027842	-0.7403264763
S6	-1.4384041930	-0.1117573618	1.0203694907
C7	-2.9104704360	-0.1539033134	-0.0734272880
H8	3.0238505371	1.4742592856	0.3267779260
H9	1.6253989506	2.5558969364	0.0322148302
H10	2.2917584882	1.5886210914	-1.2741945944
H11	-0.1807252750	-0.6583612264	-1.0874612891
H12	-0.8908865316	2.4086095428	-0.0533059870
H13	-3.7780825219	-0.2557082306	0.5784346608
H14	-2.8917475204	-0.9949639600	-0.7704056398
H15	-2.9798265199	0.7789803951	-0.6297140735
O16	0.0063789898	-1.7401312027	-1.3746067552

H17	-0.7550933222	-2.2405635218	-1.0499460028
H18	0.7616112819	-1.8158401375	-0.6064103373

18b↔19b

C1	-0.7879156537	-1.2514448324	-0.3799721492
C2	0.0643358138	-0.2326584213	0.1227011994
C3	-2.2929309943	-0.9798565394	-0.3684704045
S4	-0.3588931066	-2.9705464144	0.0083372407
O5	-0.1847980717	1.0104622783	0.0238476126
S6	1.6460120172	-0.6945045633	0.8392990860
C7	2.3589382467	0.9710019246	1.0767102262
H8	-2.5070810923	0.0764905982	-0.5411677347
H9	-2.8056372550	-1.5630707282	-1.1388940583
H10	-2.7421836857	-1.2534469133	0.5925315658
H11	0.1832266240	-3.3254177533	-1.1784504072
H12	-0.7277992382	1.4335646670	-1.1303100359
H13	3.3237192325	0.8115164645	1.5593567927
H14	2.5035240706	1.4734470830	0.1216107600
H15	1.7169588590	1.5742714831	1.7148150944
O16	-1.0426965393	1.6467074944	-2.1612435342
H17	-0.8126434978	0.6111710739	-2.6760377437
H18	-0.4903055019	2.3539454855	-2.5118280719
O19	-0.5111885830	-0.5623292507	-2.9381637478
H20	-0.5247228924	-0.9752711945	-1.9045893996
H21	-1.1876306793	-1.0157203214	-3.4537095321

19b↔20b

C1	-0.9117816890	-1.1247392713	-0.3293535466
C2	0.0738238319	-0.0946618848	0.2149263783
C3	1.1371452890	0.3054264438	-0.8387716997
S4	2.3256268704	-1.2548356000	-1.0775510926
S5	-0.8802114913	1.3875339291	0.8538385408
C6	1.9875113356	-1.6282054081	-2.8290153703
O7	0.7024300734	0.7996191496	-1.9362176338
H8	-0.3819199079	-2.0448397547	-0.5827360033
H9	-1.6792081298	-1.3556906744	0.4107719621
H10	-1.3905406125	-0.7445401226	-1.2343102265
H11	0.5765158316	-0.4689674824	1.1070956148
H12	-1.6240916734	1.5616440347	-0.2584139682
H13	1.3548291236	-2.5106406281	-2.9284509800
H14	1.4825607278	-0.7525252726	-3.2429029670
H15	2.9329496221	-1.7922796476	-3.3456421177
O16	2.1189121066	1.2136867818	-0.0821136516
H17	1.7595294247	2.6119991038	-0.0539049328
H18	2.9868416697	0.9782523583	-0.4376151611
O19	0.5717918714	3.2496844737	-2.3999251683
H20	0.6025740801	2.2156379802	-2.3061708883
H21	1.1700643867	3.5019284177	-3.1100293124

O22	1.2775558774	3.5503887474	-0.0980953922
H23	0.9663664258	3.5677790871	-1.1585437087
H24	0.4659402085	3.3417061024	0.4109589724

20b↔21b

C1	-0.0307866470	0.0491099995	-0.1917602346
C2	1.4858815902	0.1156785952	-0.3256373113
C3	-0.6905315401	1.4047088085	0.0360613679
O4	2.1701480675	-1.0228098730	-0.2275502267
O5	1.9939978752	1.0602141786	-0.9880008524
S6	2.1183161588	1.0119921802	2.1726167668
S7	-0.5531887083	-1.2486211664	1.0092092825
C8	2.9490989265	-0.4569248421	2.9041278593
H9	-0.3623246602	-0.3569997136	-1.1570520894
H10	-0.3609547260	2.1102155072	-0.7287946089
H11	-1.7748734642	1.3009963758	-0.0179056133
H12	-0.4196633605	1.8014580782	1.0149403338
H13	1.6179937655	-1.6448680608	0.2885706667
H14	3.1068663222	1.3255123227	-0.6229825139
H15	-0.0526769398	-0.5753553614	2.0764277899
H16	3.2857712691	-1.1493748076	2.1265458294
H17	3.8176499916	-0.1432280729	3.4853755055
H18	2.2694066474	-0.9868115471	3.5737942859
O19	4.0386179841	1.6623209444	0.0639947068
H20	3.5154954756	1.5387144827	0.9804895204
H21	4.7431271476	1.0028511023	0.0461924437

26↔27

C1	-0.1032824552	-1.3271354693	0.7994675155
O2	-0.6961836482	-1.6257716823	-0.3028083848
C3	0.0431038779	0.1758753348	1.0873723109
C4	1.0693180398	0.7594174746	0.1052197979
O5	0.6224763160	0.6242314030	-1.2306441111
O6	0.4993952858	0.4599083597	2.3994416615
H7	-0.2904275493	-1.9476127430	1.6900627065
H8	-0.9374341883	0.6306377261	0.8926227468
H9	1.1941384772	1.8291472795	0.3017704194
H10	0.1016995879	-0.1969379615	-1.2710967578
H11	2.0408731539	-2.5503998128	-0.8449255868
H12	-0.1198990851	0.0795906045	3.0297957852
C13	2.4082914666	0.0459320943	0.3558805924
S14	2.0857506112	-1.7358540656	0.7028617929
O15	-0.4255414499	-3.8177589897	-1.4432449346
H16	-0.6594796241	-2.9641425024	-0.8923568340
H17	-0.9925454690	-3.8104560113	-2.2210448345
O18	1.9199990947	-3.2307613459	-1.7476215591
H19	0.8768916549	-3.5642168450	-1.7010641183
H20	2.5116594209	-3.9830329425	-1.6205198193

H21	2.9031073569	0.4572562244	1.2347077479
H22	3.0512885618	0.1523587234	-0.5168947194

27↔28

C1	-0.0558299183	-0.0008381821	-0.2363195094
C2	-0.7254122969	-0.3024895447	1.0990072395
S3	-2.4721041032	-0.6137332660	0.5928781842
C4	-1.9927010167	-1.3599211712	-1.0474574642
C5	-0.5822076455	-1.0860652734	-1.1651074848
O6	-2.2091594063	-2.7716608683	-1.1417942572
O7	1.3540221119	-0.0007084647	-0.1121806239
H8	-0.4227625483	0.9718979669	-0.5945685304
H9	-0.2776794766	-1.1870677992	1.5568347048
H10	-0.6680648119	0.5373417877	1.7918306094
H11	-2.8859308317	-0.6167815787	-2.0195025141
H12	0.0607487426	-1.9645881349	-1.2066183796
H13	-3.1203172267	-2.9019455782	-1.4149694431
H14	-0.1810324899	-1.1876685448	-3.3424765618
H15	1.7172497963	0.1402395025	-0.9933793926
O16	-0.0712649951	-0.4600823600	-2.7162368942
O17	-1.5720882633	1.2823621264	-3.5478924584
H18	-1.5285435966	2.1591042169	-3.1539425904
H19	-0.7050429074	0.3641300294	-3.0680534211
O20	-3.5157743401	0.0159229221	-2.7179824949
H21	-3.9706418203	-0.5570493348	-3.3430372095
H22	-2.4913134167	0.8423083432	-3.2760975884

28↔29

C1	0.3211420866	0.5814167811	0.8997372701
C2	0.0194716022	0.6888899932	2.4132879747
S3	-1.3225196878	1.9263258621	2.6183364333
C4	-0.9951315712	2.6217867107	0.9933518846
C5	0.0291452119	1.9276809362	0.3007784779
O6	-1.6111781123	3.6655284020	0.6543532682
O7	-0.5134349135	-0.3909587537	0.2540004848
H8	1.3724424369	0.2875923716	0.7725778397
H9	-0.3003495043	-0.2692053388	2.8238824080
H10	0.8991894091	1.0370817067	2.9586522621
H11	-0.0668175458	1.9313479588	-0.7849561635
H12	-0.9634508389	4.4271154033	-0.2419066810
H13	-0.2125808142	-1.2669977244	0.5186446569
O14	1.7224226992	3.9469981915	-0.0015107302
H15	1.1528659982	3.0239039831	0.2715815789
H16	2.0613738914	4.3548927064	0.8040154370
O17	-0.2573518659	5.0285880716	-0.8337287408
H18	-0.4046134081	4.8791310358	-1.7734318707
H19	0.7840068460	4.5923186091	-0.4764678431

29↔30

C1	-2.7778782042	1.5317753426	3.3613852898
C2	-3.5711409744	2.8533378266	3.3723994591
S3	-2.3620131011	4.2394697150	3.3658493306
C4	-0.9031135681	3.1011899351	3.7877487371
C5	-1.5385020428	1.7570681122	4.2181039888
O6	0.0696103317	3.1100176833	2.9740396315
O7	-2.3367441318	1.1564143897	2.0647840622
H8	-3.4016099857	0.7358241487	3.7946846947
H9	-4.2185121546	2.9319520842	2.4960895157
H10	-4.1988379781	2.9044108695	4.2657455441
H11	-1.8372155279	1.7851259034	5.2685547769
H12	-0.8120443696	0.9550251059	4.0787121928
H13	-3.0935937555	1.1635232022	1.4713382821
O14	-0.3232419341	3.7938141246	5.1928319097
H15	-1.0313450194	3.9201020264	5.8343460300
H16	0.7859705730	3.2949160067	5.5779777940
O17	1.7883577435	1.5615513838	3.7065568608
H18	1.0134764236	2.2147149100	3.2620602748
H19	2.5477523668	1.5103646570	3.1191194915
O20	1.8116968372	2.8160743300	5.7593165913
H21	2.4674179754	3.5219905001	5.7568810673
H22	1.9433667260	2.1788578818	4.8192602477

30↔31

C1	2.3888432609	-1.6668423994	1.0832302137
O2	2.4470930596	-1.4043641076	2.3546060989
C3	1.4168667450	-1.0216996468	0.1483853664
C4	1.1784938495	0.4736572464	0.4212157214
O5	0.1811392906	0.9808337198	-0.4626846546
O6	2.8688499909	-2.8243412584	0.6800158485
C7	2.4516899083	1.2812447452	0.1954745880
S8	3.8914850727	0.4934815606	1.0110787042
H9	1.7406848206	-1.1740937913	-0.8823314999
H10	0.4714637925	-1.5679455300	0.2832199507
H11	0.8601527050	0.5928315988	1.4644727264
H12	-0.6847310114	0.7191122316	-0.1357238553
H13	3.4020686854	-3.1908851915	1.4035188908
H14	2.6491197510	1.3384822910	-0.8779270892
H15	2.3057694335	2.2979363529	0.5635503195
H16	2.9447662366	-0.4162997823	2.2714187383

27↔32

C1	-0.2014234983	0.1083168108	-0.0030849144
C2	-0.7251600809	-0.6221126048	1.2356267643
S3	-2.3105338706	-1.4537293322	0.7568663902
C4	-2.0247602990	-1.1584440292	-1.0386756124
C5	-0.5537831366	-0.7569980702	-1.2020566810

O6	-2.5627034563	-1.9463841325	-1.9135807928
O7	-0.3063324561	-0.0429937449	-2.3957031603
O8	1.1908155408	0.3156052017	0.1414894984
H9	-0.7234035363	1.0699011828	-0.1171453838
H10	-0.0016354068	-1.3800533900	1.5369827718
H11	-0.9027949287	0.0567363044	2.0676494276
H12	-2.6183819982	-0.0034849074	-1.2573574932
H13	0.0397497215	-1.6818338066	-1.1631127880
H14	-3.4277540410	-1.3524172253	-2.4197755354
H15	-0.5477458424	-0.6131165235	-3.1355314288
H16	1.5050276688	0.7054363796	-0.6826941727
C17	-3.6139740787	0.5827241582	-1.9917072108
O18	-4.0938506836	-0.3753242185	-2.7086217963
H19	-4.1888503462	0.9296814179	-1.1187301574
H20	-2.9996788562	1.3537461213	-2.4792688230

32↔33

C1	-5.7377369857	1.7413351354	-0.9990459962
S2	-3.9329742553	2.0949932765	-1.1616632031
C3	-4.0561955083	2.3828814428	-3.0854681776
C4	-5.5766551591	2.4396395455	-3.3510862160
C5	-6.2125190711	1.4153864494	-2.4083994265
O6	-3.4247841940	1.5856183530	-3.8544010486
O7	-5.8598292827	2.1474319276	-4.7007381785
O8	-7.6271352313	1.4554080533	-2.4643586180
H9	-5.8761068977	0.9076904107	-0.3123407861
H10	-6.2717336815	2.6134542112	-0.6184298499
H11	-5.9914198563	3.4273537812	-3.1204369659
H12	-5.8344595929	0.4247963450	-2.6992403568
H13	-5.0826120604	1.6458461104	-5.0019208593
H14	-7.8572415569	1.3902002532	-3.3996529813
O15	-3.4627447247	3.8379649213	-3.2655214293
H16	-2.1845481221	3.7944018730	-3.0429025416
H17	-3.8884162728	4.4059211536	-2.6091590878
O18	-0.9344203755	1.3634488656	-3.7016757811
H19	-1.9670337164	1.3084326400	-3.7655754559
H20	-0.5865559664	1.2407607770	-4.5909437890
O21	-1.0725089394	3.6396521626	-2.8720266574
H22	-0.8792666345	3.6605468898	-1.9270779796
H23	-0.8754981192	2.6247783473	-3.2543594699

33↔34

C1	-5.5669118782	2.9009092422	-1.1338760043
S2	-3.9924825820	2.1593274930	-0.5561388361
C3	-3.9126060504	1.3410282819	-3.0653736287
C4	-5.4281873745	1.5325590855	-3.2688789314
C5	-6.2676090750	1.9011443114	-2.0335566570
O6	-3.2310356847	2.3940538828	-3.5406976415

O7	-6.0174102359	0.3235029119	-3.7866567422
O8	-7.5221266132	2.4237181494	-2.4582264796
O9	-3.3626648484	0.2180114781	-3.0177557817
H10	-6.2234486400	3.1391682000	-0.2980279503
H11	-5.3615798346	3.8245471638	-1.6780865771
H12	-5.5431698474	2.3443063411	-3.9911890062
H13	-6.4252250690	0.9782398035	-1.4599598246
H14	-2.2936837018	2.2333196260	-3.3670851906
H15	-5.6864342748	0.1787674332	-4.6767625264
H16	-7.9535977864	1.7389926443	-2.9828592524
H17	-4.1094870184	-0.7641695522	-2.4378169532
O18	-4.7840608158	-1.4661261064	-1.9549490768
H19	-5.6115978982	-1.3124512383	-2.4349199386
H20	-4.9055231749	-1.1176031713	-0.9284704312
O21	-5.0568422618	-0.5029742501	0.2689519961
H22	-4.6292301510	-0.8863026909	1.0401498010
H23	-4.6769021712	0.4524894392	0.1236216976
